# Comprehensive dietary patterns explain acquired cystic kidney disease risk through genetic and metabolomic mechanisms

**DOI:** 10.3389/fnut.2025.1611656

**Published:** 2025-10-29

**Authors:** Chenhao Xu, Junjie Zhao, Kan Wu, Shengzhuo Liu, Fan Zhang, Qin Cao, Zhouwei Wan, Yongquan Tang, Zhihong Liu, Hao Zeng, Xianding Wang, Jiayu Liang

**Affiliations:** ^1^Department of Urology, Institute of Urology, Sichuan Clinical Research Center for Kidney and Urologic Diseases, West China Hospital, Sichuan University, Chengdu, China; ^2^School of Biomedical Sciences, The Chinese University of Hong Kong, Shatin, Hong Kong SAR, China; ^3^Department of Urology, Institute of Urology, Kidney Transplant Center, West China Hospital, Sichuan University, Chengdu, Sichuan, China; ^4^Department of Pediatric Surgery, West China Hospital, Sichuan University, Chengdu, China; ^5^State Key Laboratory of Biotherapy and Cancer Center, West China Hospital, Sichuan University, Chengdu, China

**Keywords:** renal cyst, diet, reduced rank regression, kidney function, CDPI

## Abstract

**Background:**

Acquired renal cysts (ARC) are associated with kidney function decline, necessitating novel dietary pattern (DP) analyses in large cohorts.

**Methods:**

This UK Biobank prospective cohort study (2006–2010) included participants with ≥2 dietary records, excluding those with severe kidney damage. The constructed comprehensive dietary pattern integration (CDPI) utilized reduced rank regression (RRR) and latent profile analysis (LPA). ARC cases (ICD-10: N28.1) were assessed via Cox regression for risk and dose–response, with NMR metabolites examined as mediators.

**Results:**

Among 119,709 participants (median follow-up: 10.57 years), 850 ARC cases were identified. Lipid-rich and hyperglycemic diets increased ARC risk [e.g., HRs for G1.DP1: 1.080 (1.024, 1.139); G1.DP2: 1.144 (1.048, 1.249)], while micronutrient-rich diets showed weak protective effects [G4.DP1: 0.943 (0.892, 0.998)]. LPA confirmed RRR findings, and 7/251 NMR metabolites had significant mediating effects.

**Conclusion:**

Diets high in fat (cheese, butter, pizza) and sugar (chocolate, sugary drinks) elevated ARC risk, whereas micronutrient- and fiber-rich diets (vegetables, fruit, lean poultry, nuts, eggs) were protective. Key mediators included branched-chain amino acids, IGF-1, and RBC distribution width.

## Introduction

Acquired renal cysts (ARC) are a prevalent renal condition, primarily characterized by benign cystic structures (1). Detection has markedly increased in routine physical examinations. Several cohort studies suggest that renal cysts may impair renal function and increase the risk of nephron loss (2–4). Additionally, ARC is associated with a higher risk of metabolic syndrome, particularly hypertension, which is an independent risk factor (3, 5, 6). Despite the high prevalence of ARC, research on its etiology and management is limited, with minimal attention given to potential dietary influences (7–9). Both healthy individuals and patients with ARC express a strong desire for dietary guidance (10).

Most research on diet and renal cysts has concentrated on polycystic kidney disease (PKD), a genetic disorder due to its significant clinical features and genetic basis (11). For example, high sodium intake has been linked to accelerated cyst growth in PKD, likely due to increased vasopressin levels (12, 13). Animal studies have also shown that plant-based proteins reduce kidney weight and cyst size in PKD models (14), and calorie restriction may inhibit cyst growth. Furthermore, increased consumption of fruits, vegetables, and adherence to a Mediterranean diet has been associated with better renal health (15).

Reduced rank regression (RRR) is a data-driven method that utilizes prior knowledge to identify dietary patterns by exploring how specific nutrients associated with causal pathways relate to disease outcomes (16). Latent Profile Analysis (LPA) is able to classify individuals into dietary subgroups based on multiple dietary variables and revealing inherent patterns within the population (17). Using RRR and LPA, we developed a comprehensive dietary pattern integration (CDPI) framework to capture dietary patterns comprehensively. Both RRR and LPA have been widely used in studies examining the impact of dietary patterns on disease outcomes (18, 19).

In this study, we used detailed dietary data from UK Biobank (UKB) participants to identify dietary patterns (DPs), assess their variability, and evaluate their association with the risk of ARC.

## Methods

### Study population

The UKB is a population-based cohort consisting of over 500,000 participants aged 37 to 73 from 22 locations across England, Wales, and Scotland. Baseline data were collected between 2006 and 2010 and linked to hospital and mortality records. Detailed sociodemographic, health behavior, and medical history information was gathered through touchscreen questionnaires and interviews. Physical measurements and biological samples were collected by trained staff following standardized protocols. Written informed consent was obtained from all participants (20, 21).

### Dietary intake measurement

The Oxford WebQ, a web-based dietary instrument, was used to capture extensive dietary data from participants (22). This tool, validated against an interviewer-administered 24-h recall questionnaire, recorded the consumption of up to 206 food items and 32 beverage types from the previous day (22). Participants with valid email addresses completed the questionnaire at baseline and during four intervals between April 2009 and June 2012. Only participants with at least two completed assessments were included in the analysis, and their average dietary intake was calculated.

Following established procedures (23), dietary information was categorized into 50 primary food groups (Supplementary Table S1), aligned with the U.K. National Diet and Nutrition Survey. Nutritional and energy intake was calculated by multiplying each food portion by its nutrient composition using data from the UK Nutrient Databank (2012–2014) (23). Energy density (kJ/g) was calculated by dividing total energy by the total weight of food (excluding beverages) (24). The percentage of energy from saturated fatty acids (SFA) and free sugars was calculated by dividing energy from SFA/free sugars by total daily energy intake. Fiber density (g/MJ) was assessed by dividing daily fiber intake by total energy intake, multiplied by 1,000. Definitions for free sugars and fiber followed U.K. Scientific Advisory Committee on Nutrition guidelines, with fiber measured using the Englyst method (25).

To address dietary misreporting, the ratio of energy intake (EI) to estimated energy requirements (EER) was calculated using the Schofield equation (Supplementary Note 1), based on the 1985 FAO/WHO/UNU Report on Human Energy Requirements (26, 27). Participants classified as dietary under-reporters (EI <95% CI) or over-reporters (EI >95% CI) were excluded (28).

### Measurement of metabolic biomarkers

Between June 2019 and June 2022, metabolic biomarkers were measured using a high-throughput nuclear magnetic resonance (NMR) platform developed by Nightingale Health Ltd. A total of 251 biomarkers, including lipoprotein lipids across 14 subclasses, fatty acids, and low-molecular-weight metabolites, were analyzed from EDTA plasma samples of approximately 280,000 participants. Detailed methods and biomarker data have been previously published (28).

### Outcome ascertainment

The UKB dataset includes “first occurrence” fields that map clinical codes from primary care visits, inpatient admissions, death records, and self-reported medical conditions to ICD-9 and ICD-10 codes. The outcome of interest in this study was “Cyst of kidney, acquired,” identified using ICD-10 code N28.1 and ICD-9 code 593.2. Data were censored at the earliest of three events: the first diagnosis of an ARC, participant death, or the data cutoff (October 31, 2022, for HES data; August 31, 2022, for SMR; and May 31, 2022, for PEDW) (29).

### Covariates

Data on age, gender, ethnicity, educational level, household income, alcohol consumption, physical activity, smoking status, depression scores, health scores, baseline renal function, sleep duration, and medical history (focusing on cardiovascular diseases, diabetes, and kidney diseases) were collected through questionnaires administered via touchscreen computers, additional health records and other biochemical tests results. Sleep duration was assessed by asking, “How many hours do you sleep per 24-h period?” and calculating the average across multiple surveys. Physical activity was evaluated using the short form of the International Physical Activity Questionnaire (IPAQ). Detailed information on the variables, along with a display of the directed acyclic graph illustrating the relationships between them, can be found in the Supplementary Figure S1.

### Genetic risk scores

We utilized summary-level GWAS data from the tenth data release of the FinnGen database (Cyst of kidney, XIV Diseases of the genitourinary system (N14), cases: 1,874, controls: 408,319) to compute PRS for ARC (30). PRS was calculated using PRSice-2, with *p*-value thresholds (e.g., *p* < 5 × 10^−8^, *p* < 1 × 10^−5^) to ensure robustness. Linkage disequilibrium (LD) clumping (*R*^2^ < 0.1, window size 250 kb) was used to reduce redundancy. Quality control included excluding SNPs with minor allele frequency (MAF) < 0.01 and genotype missingness >5%. PRS model performance was evaluated using the C-statistic and AUC, and the PRS was included in a Cox proportional hazards model to account for genetic predispositions (31, 32).

### Comprehensive dietary pattern integration framework

Our CDPI framework combines data-driven DP identification with health-specific nutrient selection to examine the relationship between DPs and health outcomes, specifically ARC. The framework follows five key stages:

**Stage 1: Data-driven dietary pattern construction using RRR**

We initiated the CDPI framework by constructing DPs through RRR. RRR identifies linear combinations of food groups correlated with these pre-selected nutrient response variables, aiming to maximize the explained variance.

**Stage 2: Health-focused nutrient selection**

To further refine the DPs, we conducted a systematic review of existing literature on DPs and health outcomes, consulting nutritional experts to define nine health-specific focus areas: basic nutrition and energy balance, cardiovascular health, bone health, antioxidation, glycemic control, iron metabolism, blood pressure regulation, renal protection, and metabolic health. Each of these areas is crucial for understanding their multifaceted influences on renal cyst formation and overall kidney health (33–36). For each health focus, we selected relevant nutrients implicated in disease pathways and performed RRR on data derived from 50 food groups (nutrient combinations are detailed in Supplementary Table S2) (37). DPs were converted into *Z*-scores, reflecting each participant’s adherence to specific DPs. Food groups with higher factor loadings had a more significant influence on the identified patterns, and only DPs that explained more than 15% of the variance within each health focus were included in further analysis.

**Stage 3: Synthesis and robustness enhancement**

After the initial RRR-based analysis, we conducted secondary analyses to enhance the robustness of the results. We extracted the food groups with the highest factor loadings across the nine health-focused DPs. We implemented a pre-specified, reproducible workflow. First, each DP was represented by its standardized 50-food-group loading vector; top-loading sets were defined by |loading| ≥0.20 with sign preserved. Second, pairwise DP similarity combined three metrics—Pearson correlation of full loading vectors, Jaccard overlap of signed top-loading sets, and cosine similarity—into a composite score *S* (mean of the three). Third, hierarchical agglomerative clustering (average linkage) on 1 − *S* determined the number of clusters by maximizing average silhouette width (target 4–6 clusters). Fourth, robustness was examined by 200 bootstrap resamples (refitting RRR and repeating the pipeline); stability was summarized by the adjusted Rand index (ARI). Finally, clusters were labeled by dominant signed loadings (food-group anchors and nutrient themes). Two independent nutrition epidemiologists applied a predefined rubric to confirm membership and assign labels; agreement was quantified by Cohen’s *κ*, with discrepancies adjudicated by a senior reviewer.

The final synthesized DPs identified through this process included: lipid-rich, calorically dense diets; hyperglycemic, fiber-deficient diets; micronutrient-abundant, low-lipid diets; mineral-rich, moderate-fat diets; and fiber-enriched, lipid-conservative diets. Detailed variance explanations for each DP can be found in Supplementary Tables S3–S11.

**Stage 4: Prospective association of DPs with ARC**

The prospective association between the synthesized DPs and ARC risk was examined using multivariable Cox proportional hazards models, adjusted for a comprehensive set of covariates that is listed above. Hazard ratios (HRs) and 95% confidence intervals (CIs) were calculated for each unit increase in DP *Z*-scores. DPs were included as both continuous variables (using *Z*-scores) and categorical variables (divided into quartiles, with the lowest quartile as the reference group). Restricted cubic spline models were used to examine non-linear associations between DP *Z*-scores and ARC incidence, adjusting for the same set of covariates.

Likelihood ratio tests were performed to assess heterogeneity in the relationship between DPs and the risk of ARC across different age groups (<50 years, 50–60 years, >60 years), genders (female, male), smoking statuses (never, previous, current), physical activity levels (low, moderate, high), and BMI categories (Q1–Q4).

**Stage 5: Dietary profile analysis**

We employed finite normal mixture modeling, using the R package “mclust,” to empirically identify subgroups with similar DPs based on data from 50 food groups. This approach assumes that the data are generated from a mixture of normal distributions, each representing a distinct subgroup. To select the best-fitting model, we evaluated multiple configurations using the Bayesian information criterion, integrated completed likelihood, and bootstrap likelihood ratio test (38, 39).

After identifying the optimal model, we characterized each subgroup based on covariates and dietary intake patterns, revealing specific dietary profiles. These dietary profiles were then incorporated as categorical variables into subsequent statistical models. To examine the association between naturally occurring dietary subgroups and ARC, we used each dietary profile sequentially as a reference group in the Cox proportional hazards models. The models were adjusted for potential confounders, allowing us to assess the relative risk of ARC across different dietary subgroups while controlling for all relevant covariates.

### Mediation effect analysis of NMR metabolites

We utilized the mediation package in R to establish a standard three-variable path model to assess the mediation effects of NMR metabolites on the associations between DPs, represented by the *Z*-scores derived from participants’ corresponding RRR, and the risk of ARC (40). Linear regression models were employed to analyze the relationship between DPs and metabolites, while COX regression models were applied for the metabolite-ARC association. The same covariates used in the initial association analyses were included in this model. The significance of the mediation effects was determined through 2,000 bootstrap iterations, ensuring robust statistical inference.

### Sensitivity and subgroup analysis

We conducted several sensitivity analyses, beginning with the exclusion of participants who developed ARC within 2 years following their most recent 24-h online dietary assessment. Baseline metabolic syndrome was defined according to the harmonized criteria established by the International Diabetes Federation and the American Heart Association/National Heart, Lung, and Blood Institute in 2009 and included as a covariate in our analyses. Additionally, we excluded individuals with baseline renal dysfunction (eGFR <60 mL/min/1.73 m^2^). Furthermore, we performed stratified analyses considering various covariates, including gender, age, Townsend deprivation index, education level, BMI, smoking status, alcohol consumption, IPAQ, diabetes, cardiovascular disease, and hypertension.

All analyses were conducted using R version 4.3.1, and the epidemiological study adhered to the STROBE guidelines.

## Results

### Population characteristics

Out of the 502,186 participants recruited in the UKB study, we applied several exclusion criteria. These included individuals who did not complete any validated dietary assessments (*n* = 273,383) or who completed only one 24-h online dietary assessment (*n* = 79,693). Additionally, participants lacking genomic data necessary for calculating PRS for ARC (*n* = 32,351) were excluded. We also excluded participants with ARC diagnoses recorded prior to baseline (ICD-10 code N28.1, *n* = 1,082), those missing specific nutrient data (*n* = 18), and those with extreme energy intake values based on the ratio of EI to EER (over-reporters: *n* = 28, under-reporters: *n* = 1,223). Further exclusions were made for individuals with baseline cancer (*n* = 6,217), end-stage renal disease (*n* = 9), those undergoing renal replacement therapy (*n* = 2), a history of kidney surgeries (*n* = 4), frequent urinary tract infections (*n* = 32), long-term use of nephrotoxic medications (*n* = 15), or PKD (*n* = 24).

**Figure 1:**
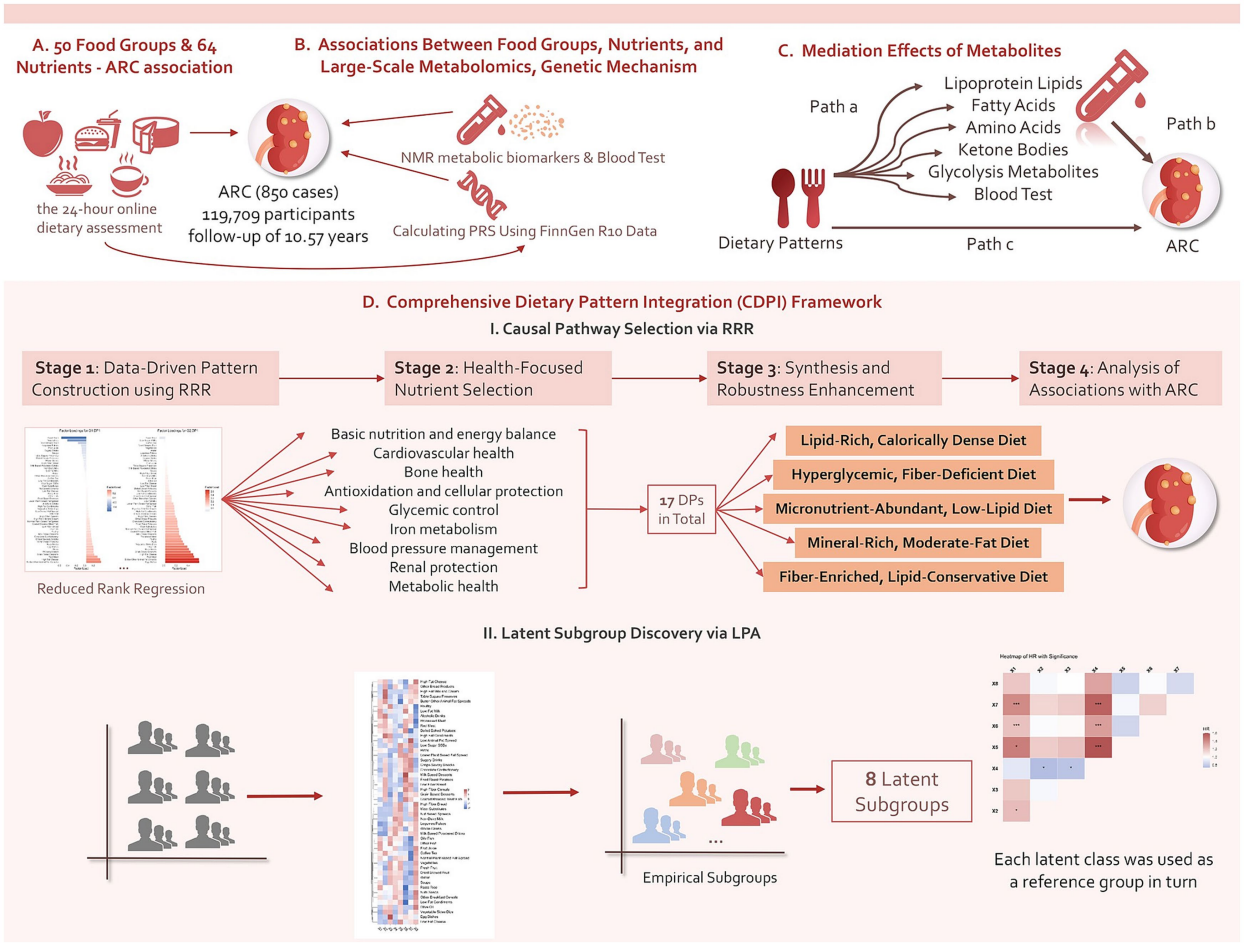
Summary of the analyses conducted in this study. Using data from the UKB, we initially conducted an exploratory investigation into the prospective associations of 50 food groups and 64 nutrients with ARC events and levels of 215 metabolites measured by the NMR platform, adjusting for confounders. Additionally, we incorporated PRS data as covariates to examine the role of genetic factors and further explored the mediating effects of certain associated metabolites. We then developed a CDPI framework to synthesize five DPs and analyze their prospective associations with ARC, dose–response relationships, and the mediating effects of NMR metabolites, enhancing robustness through stratified analyses. Finally, the study also examined potential dietary profiles in the general population and their associations with ARC.

Ultimately, 119,709 participants were included in the final analysis, having completed at least two 24-h online dietary assessments and provided genomic data for PRS calculations. The average follow-up period was 10.57 years, during which 850 ARC cases were documented. Figure 1 summarizes the overall analytical approach used in this study.

Table 1 presents the baseline demographic, socioeconomic, and clinical characteristics of the study participants, categorized by those who developed ARC and those who did not. Participants who developed ARC were more likely to be older males, have lower socioeconomic status, lower education levels, and higher rates of smoking and alcohol consumption. These individuals also had higher prevalence rates of obesity, cardiovascular disease, hypertension, and diabetes compared to participants who did not develop ARC.

**Table 1:** Baseline characteristics of participants by outcomes (*N* = 119,709).

Characteristics	Overall	Acquired cystic kidney disease		
	*N* = 119,709	No (*N* = 118,859)	Yes (*N* = 850)	*p* ^a^
Male, *n* (%)	53,169 (44.4)	52,646 (44.3)	523 (61.5)	<0.001
TDI^b^	−1.64 (2.84)	−1.64 (2.84)	−1.29 (3.00)	<0.001
Age (years)^b^	56.12 (7.83)	56.09 (7.83)	60.36 (6.60)	<0.001
Education score^b^	11.35 (13.48)	11.34 (13.47)	12.95 (14.64)	0.001
Employment score^b^	0.78 (3.69)	0.78 (3.68)	0.76 (4.67)	0.88
Health score^b^	0.17 (3.98)	0.17 (3.96)	0.65 (5.66)	<0.001
Sleep duration^b^	7.17 (0.96)	7.17 (0.96)	7.19 (1.18)	0.589
BMI^b^	26.71 (4.57)	26.70 (4.56)	28.32 (5.18)	<0.001
Ethnicity white, *n* (%)	115,657 (96.6)	114,838 (96.6)	819 (96.4)	0.742
BMR^b^	1537.32 (259.75)	1536.69 (259.49)	1625.69 (280.29)	<0.001
Overall health rating, *n* (%)				<0.001
Excellent	25,840 (21.6)	25,749 (21.7)	91 (10.7)	
Fair	19,079 (15.9)	18,877 (15.9)	202 (23.8)	
Good	71,414 (59.7)	70,911 (59.7)	503 (59.2)	
Poor	3,145 (2.6)	3,093 (2.6)	52 (6.1)	
Smoking status, *n* (%)				<0.001
Never	42,886 (35.8)	42,500 (35.8)	386 (45.4)	
Previous	68,472 (57.2)	68,084 (57.3)	388 (45.6)	
Current	8,351 (7.0)	8,275 (7.0)	76 (8.9)	
Alcohol drinker status, *n* (%)				<0.001
Never	3,466 (2.9)	3,420 (2.9)	46 (5.4)	
Previous	112,832 (94.3)	112,064 (94.3)	768 (90.4)	
Current	3,411 (2.8)	3,375 (2.8)	36 (4.2)	
Physical activity (IPAQ), *n* (%)				0.002
Low	50,453 (42.1)	50,096 (42.1)	357 (42.0)	
Moderate	47,128 (39.4)	46,829 (39.4)	299 (35.2)	
High	22,128 (18.5)	21,934 (18.5)	194 (22.8)	
Cardiovascular disease, *n* (%)	34,753 (29.0)	34,191 (28.8)	562 (66.1)	<0.001
Hypertension, *n* (%)	32,196 (26.9)	31,675 (26.6)	521 (61.3)	<0.001
Diabetes, *n* (%)	7,715 (6.4)	7,551 (6.4)	164 (19.3)	<0.001
Nutrients intake				
Energy intake (MJ/day)^b^	7342.42 (1950.33)	7342.13 (1949.49)	7381.99 (2066.42)	0.553
Energy density (kJ/g)^b^	6.46 (1.47)	6.46 (1.47)	6.58 (1.53)	0.018
Englyst fiber (g/day)^b^	17.94 (5.94)	17.94 (5.94)	17.68 (5.96)	0.203
Free sugar (g/day)^b^	60.12 (31.78)	60.09 (31.75)	63.97 (35.88)	<0.001
Protein (g/day)^b^	80.82 (21.03)	80.82 (21.03)	81.15 (21.53)	0.649
Saturated fatty acids (g/day)^b^	27.26 (10.69)	27.26 (10.69)	28.07 (11.59)	0.027
Sodium (g/day)^b^	1963.68 (675.46)	1963.32 (675.34)	2014.04 (689.46)	0.029
Main food groups (g/day)				
High fat cheese^b^	14.74 (16.45)	14.73 (16.44)	15.92 (17.76)	0.036
Red meat^b^	39.43 (42.40)	39.40 (42.37)	43.73 (45.85)	0.003
Vegetables^b^	188.89 (135.08)	188.96 (135.11)	179.82 (131.31)	0.049
Fresh fruit^b^	195.48 (145.76)	195.52 (145.74)	188.99 (148.87)	0.193
Whole grains^b^	8.51 (27.01)	8.53 (27.04)	6.01 (21.90)	0.007
Coffee tea^b^	800.01 (325.56)	800.21 (325.56)	773.30 (324.87)	0.016
Alcoholic drinks^b^	257.84 (375.43)	257.76 (375.20)	268.35 (407.07)	0.413
SSBs and other sugary drinks^b^	69.44 (176.87)	69.44 (176.79)	69.83 (187.81)	0.948
Water^b^	510.40 (374.25)	510.66 (374.39)	474.15 (353.49)	0.005
High fat condiments^b^	13.49 (15.54)	13.49 (15.54)	13.74 (16.38)	0.636
Milk based desserts^b^	24.55 (38.70)	24.52 (38.67)	27.57 (42.05)	0.022
Chocolate confectionery^b^	11.84 (19.47)	11.84 (19.46)	11.77 (20.98)	0.92
Butter and other animal-fat spreads^b^	5.15 (8.27)	5.14 (8.26)	5.76 (9.11)	0.029

TDI, Townsend deprivation index; IPAQ, International Physical Activity Questionnaire; SSBs, sugar-sweetened beverages; BMR, basal metabolic rate.

a ANOVA or *χ*^2^ test where appropriate.

b Mean (SD).

In terms of dietary intake, total energy consumption did not differ significantly between the two groups. However, participants who developed ARC had higher energy density in their diets, characterized by increased intake of free sugars, SFA, and sodium. Specific food groups contributing to this higher energy density included high-fat cheese, red meat, milk-based desserts, and butter or animal-fat spreads. Conversely, their intake of vegetables, whole grains, caffeinated beverages, and water was notably lower than that of participants without ARC.

### Prospective associations between food groups, nutrients, biomarkers, and ARC

The exploratory analysis results, including each of the 50 food groups and 64 nutrients as single independent variables (adjusting for all covariates), are presented in Supplementary Tables S12, S13. We found that sugary drinks, high-fat cheese, and preserved sugars have a significant positive effect on the incidence of ARC, while fresh fruit and poultry have a negative effect. Among the nutrients, magnesium, pantothenic acid, vitamin B6, niacin equivalent, and biotin showed protective effects against the disease, with the effects of fats and sugars remaining significant in the nutrient analysis. The role of genes (interaction of PRS as a covariate) did not appear to be significant in any of the analyses.

We conducted multivariate logistic regression using 50 food groups, 64 nutrients, and the 17 comprehensive DPs obtained from the CDPI Framework as independent variables against 310 biomarkers and biochemical indicators. The majority of dietary factors were found to be associated with changes in the metabolome, with the results presented in the Supplementary Table.

### Comprehensive DP integration framework

Based on the RRR model, we derived 43 DPs across nine selected groups (G1–G9). We synthesized 17 RRR-derived patterns into five dietary habits using an objective clustering framework (overall silhouette 0.60; median bootstrap ARI 0.78), with excellent inter-rater agreement on labels (*κ* 0.86). Out of these, 17 DPs with an average explained variance exceeding 15% were included in the analysis. These patterns are specifically G1.DP1 (47.22%), G1.DP2 (15.69%), G2.DP1 (44.66%), G2.DP2 (15.13%), G3.DP1 (46.01%), G3.DP2 (16.93%), G4.DP1 (37.12%), G4.DP2 (22.85%), G5.DP1 (51.33%), G5.DP2 (23.61%), G6.DP1 (39.82%), G6.DP2 (19.49%), G7.DP1 (48.95%), G7.DP2 (22.80%), G8.DP1 (54.99%), G8.DP2 (22.03%), and G9.DP1 (66.53%). These patterns collectively achieved cumulative explained variances of 60.9, 59.79, 62.94, 59.97, 74.49, 59.31, 71.75, 77.02, and 66.53% for each nutrient group, respectively.

In secondary analyses of these 17 DPs, we focused on the food groups with the highest and lowest factor loadings for each pattern, as detailed in Supplementary Tables S3–S11. Overall model factor loadings are depicted in Supplementary Tables S3–S7 Supplementary Figures S3–S7. By merging and extracting prominent features from similar DPs, we identified five typical dietary habits: a lipid-rich, calorically dense diet encompassing G1.DP1, G2.DP1, G2.DP2, G3.DP2, G7.DP2, and G8.DP2; a hyperglycemic, fiber-deficient diet including G1.DP2, G5.DP1, G5.DP2, and G6.DP2; a micronutrient-abundant, low-lipid diet comprising G4.DP1, G4.DP2, and G6.DP1; a mineral-rich, moderate-fat diet consisting of G3.DP1, G7.DP1, and G8.DP1; and a fiber-enriched, lipid-conservative diet represented by G9.DP1.

### Relationships between DPs and ARC

**Figure 2:**
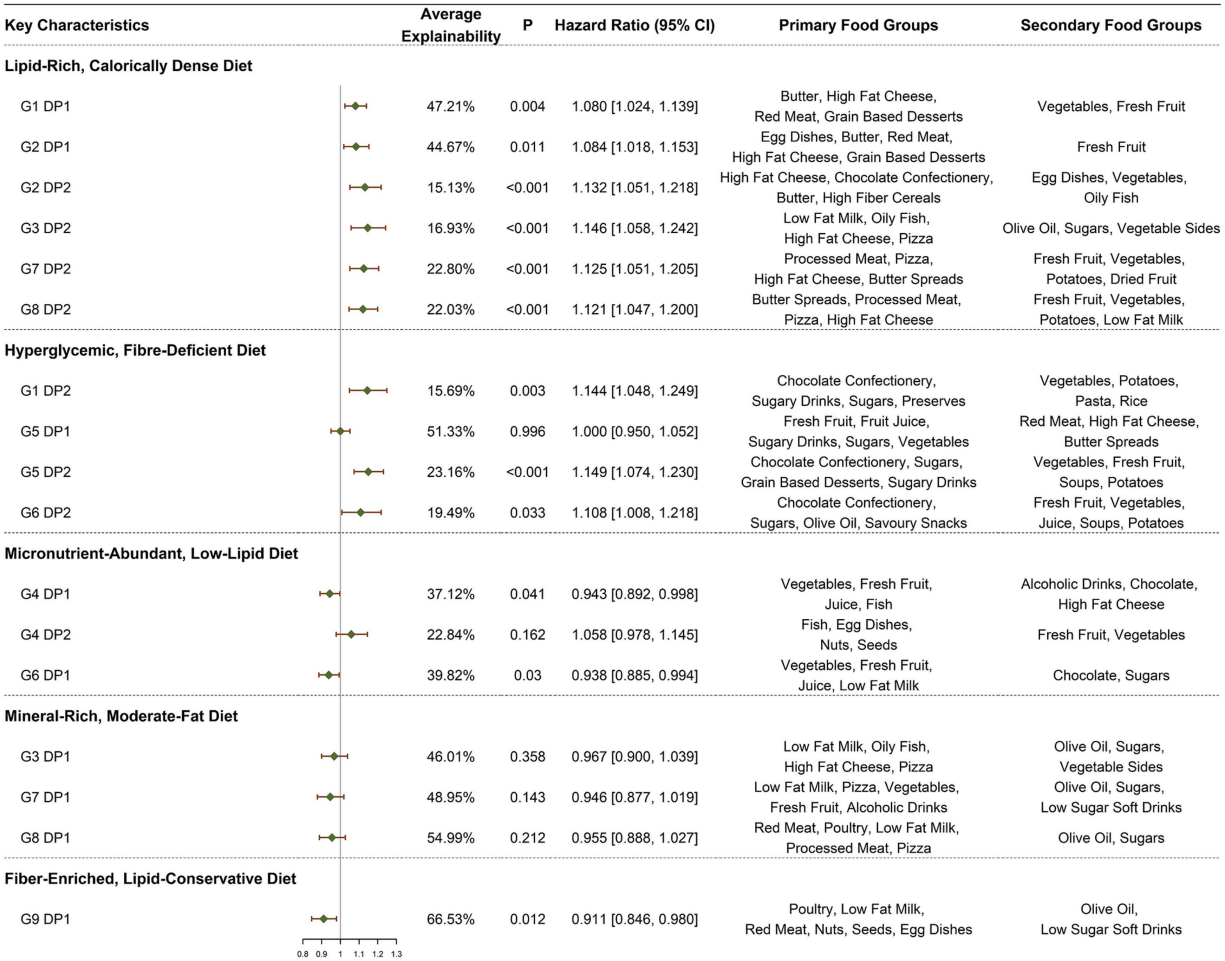
HRs (95% CI) for ARC symptoms by DPs. All models adjusted for demographic and socio-economic covariates, including gender, age at recruitment, socio-economic status (Townsend deprivation index), employment status, educational attainment, health status, baseline renal function, and log-transformed total caloric intake, lifestyle and health conditions, including average hours of sleep per night, current smoking status, BMI, current alcohol consumption status, IPAQ, PRS, heart disease, hypertension, and diabetes.

As illustrated in Figure 2, our initial approach involved incorporating the *Z*-scores of 17 DPs as continuous variables into the model, with subsequent analyses adjusted for all covariates.

In the context of a lipid-rich, calorically dense diet, six DPs consistently demonstrated a positive association with the incidence of ARC, with HRs (95% CIs) as follows: G1.DP1 [1.080 (1.024, 1.139), *p* = 0.004], G2.DP1 [1.084 (1.018, 1.153), *p* = 0.011], G2.DP2 [1.132 (1.051, 1.218), *p* < 0.001], G3.DP2 [1.146 (1.058, 1.242), *p* < 0.001], G7.DP2 [1.125 (1.051, 1.205), *p* < 0.001], and G8.DP2 [1.121 (1.047, 1.200), *p* = 0.001]. Within the dietary habits characterized by a hyperglycemic, fiber-deficient diet, a strong positive correlation was observed in all but G5.DP1, with significant associations noted in G1.DP2 [1.144 (1.048, 1.249), *p* = 0.003], G5.DP2 [1.149 (1.074, 1.230), *p* < 0.001], and G6.DP2 [1.108 (1.008, 1.218), *p* = 0.033]. Conversely, in the context of a micronutrient-abundant, low-lipid diet, except for G4.DP2, the remaining sources of DPs demonstrated a weakly inverse relationship with ARC, notably G4.DP1 [0.943 (0.892, 0.998), *p* = 0.041] and G6.DP1 [0.938 (0.885, 0.994), *p* = 0.030]. For DPs described as mineral-rich, moderate-fat diets, no statistically significant correlations with the onset of ARC were found, although the effects were generally inversely related. Specifically, G9.DP1, representing a fiber-enriched, lipid-conservative diet, showed a strong negative correlation with ARC, G9.DP1 [0.911 (0.846, 0.980), *p* = 0.012].

We further categorized the 17 DPs into quartiles based on their *Z*-scores, using the first quartile as the reference group for model construction, with adjustments made for all covariates. The DPs that showed statistically significant differences include G1.DP1 Q4 [1.267 (1.039, 1.546), *p* = 0.019, *p* for trend = 0.007], G1.DP2 Q4 [1.215 (1.005, 1.470), *p* = 0.044, *p* for trend = 0.033], G2.DP2 Q4 [1.250 (1.033, 1.512), *p* = 0.022, *p* for trend = 0.006], G3.DP2 Q4 [1.365 (1.121, 1.663), *p* = 0.002, *p* for trend = 0.001], G4.DP1 Q4 [0.811 (0.663, 0.991), *p* = 0.040, *p* for trend = 0.026], G4.DP2 Q4 [1.261 (1.001, 1.588), *p* = 0.049, *p* for trend = 0.036], G5.DP2 Q4 [1.289 (1.055, 1.576), *p* = 0.013, *p* for trend = 0.002], G6.DP1 Q3 [0.730 (0.593, 0.899), *p* = 0.003, *p* for trend = 0.003], G7.DP2 Q4 [1.377 (1.130, 1.677), *p* = 0.001, *p* for trend <0.001], and G8.DP2 Q4 [1.340 (1.105, 1.625), *p* = 0.003, p for trend <0.001]. For more detailed information, see Supplementary Table S15.

**Figure 3:**
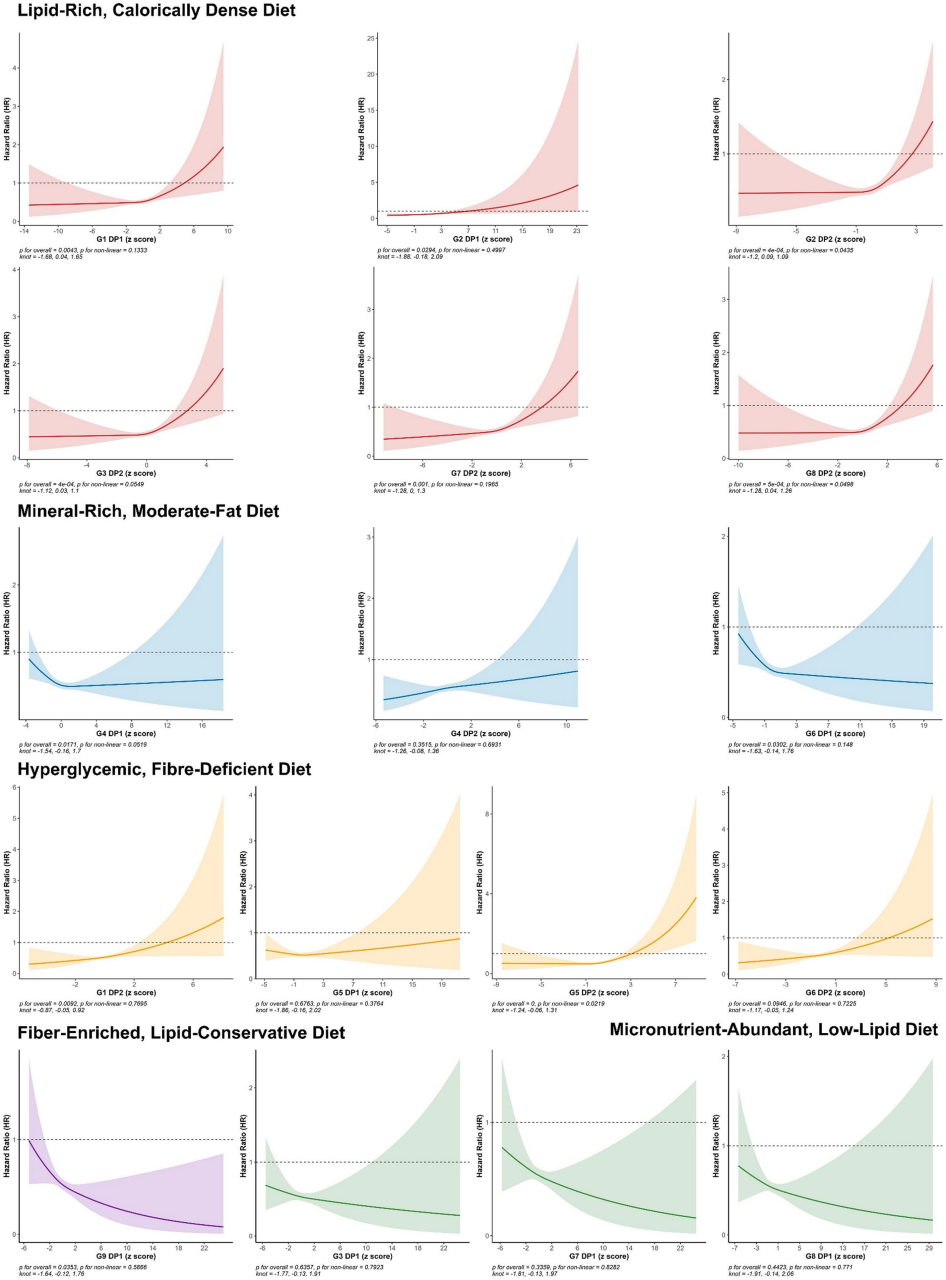
HRs (95% CIs) of continuous DP *Z*-scores for the risk of ARC. All models adjusted for demographic and socio-economic covariates, including gender, age at recruitment, socio-economic status (Townsend deprivation index), employment status, educational attainment, health status, base line renal function, and log-transformed total caloric intake, lifestyle and health conditions, including average hours of sleep per night, current smoking status, body mass index (BMI), current alcohol consumption status, physical activity level (IPAQ), PRS (PRS), heart disease, hypertension, and diabetes.

In the dose–response analysis using restricted cubic splines (Figure 3), apart from G2.DP2, G8.DP2, and G5.DP2, which showed a nonlinear association (non-linear *p* < 0.05), the nonlinear relationships for other DPs were not significant.

In summary, poor DPs high in sugar, fat, and low in fiber are significantly associated with increased risk of ARC. Conversely, micronutrient-rich and high-fiber diets are linked to a reduced risk of ARC, primarily in a linear manner.

In the subgroup analysis (conducted on 11 variables, with results detailed in Supplementary Tables S16–S26), several insightful results were identified. For instance, lipid-rich, calorically dense diets and hyperglycemic, fiber-deficient diets showed consistently strong associations in men but not in women. These diets also had a strong positive effect in the lowest (Q1) and highest (Q4) BMI quartiles but not in the Q2 and Q3 quartiles. Although mineral-rich, moderate-fat diets were not statistically significant in the overall analysis, they showed a consistent protective effect in individuals aged 50–60. Additionally, the positive promoting effects of the two aforementioned unhealthy DPs were more pronounced in individuals with a history of smoking and those who currently consume alcohol. Compared to individuals with high or low levels of physical activity, those with moderate physical activity did not seem to be affected by unhealthy DPs. In the sensitivity analysis, excluding participants who developed renal cysts within 2 years resulted in stronger associations. Adjusting for MetS led to a weakened association between all DPs and ARC risk. After excluding individuals with abnormal eGFR, the correlations for lipid-rich, calorically dense and hyper glycemic, fiber-deficient diets with ARC risk were attenuated, while the previously non-significant association for the mineral-rich, moderate-fat diet became slightly evident.

**Figure 4:**
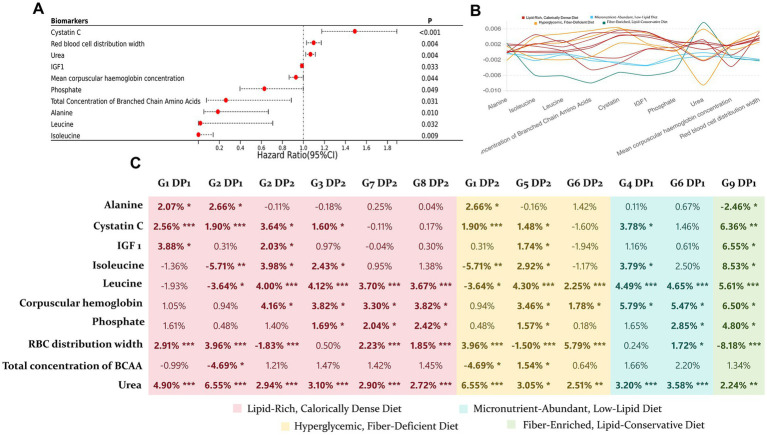
Mediation effects of NMR platform biomarkers on the impact of 12 DPs on ARC. **(A)** Cox regression analysis was performed on biomarkers and ARC, adjusting for all covariates, to identify 10 biomarkers with independent effects. **(B)** Line plot showing the magnitude of mediation effects of 10 biomarkers on the 12 DPs. **(C)** Proportion of each mediation effect, with significance levels indicated by asterisks: mediation effect *p* < 0.005 as “***,” 0.005 < *p* < 0.01 as “**,” and 0.01 < *p* < 0.05 as “*”.

We adopted a mediation analysis on 12 out of the 17 DPs that showed independent effects (a mineral-rich, moderate-fat diet DP was not included as it was not statistically significant). The potential mediators were biomarkers that had independent effects on ARC (Figure 4), totaling 10 biomarkers. Among these, seven biomarkers exhibited partial but significant mediation effects across different DPs, independent of covariates.

### Dietary profiles and association with ARC

**Figure 5:**
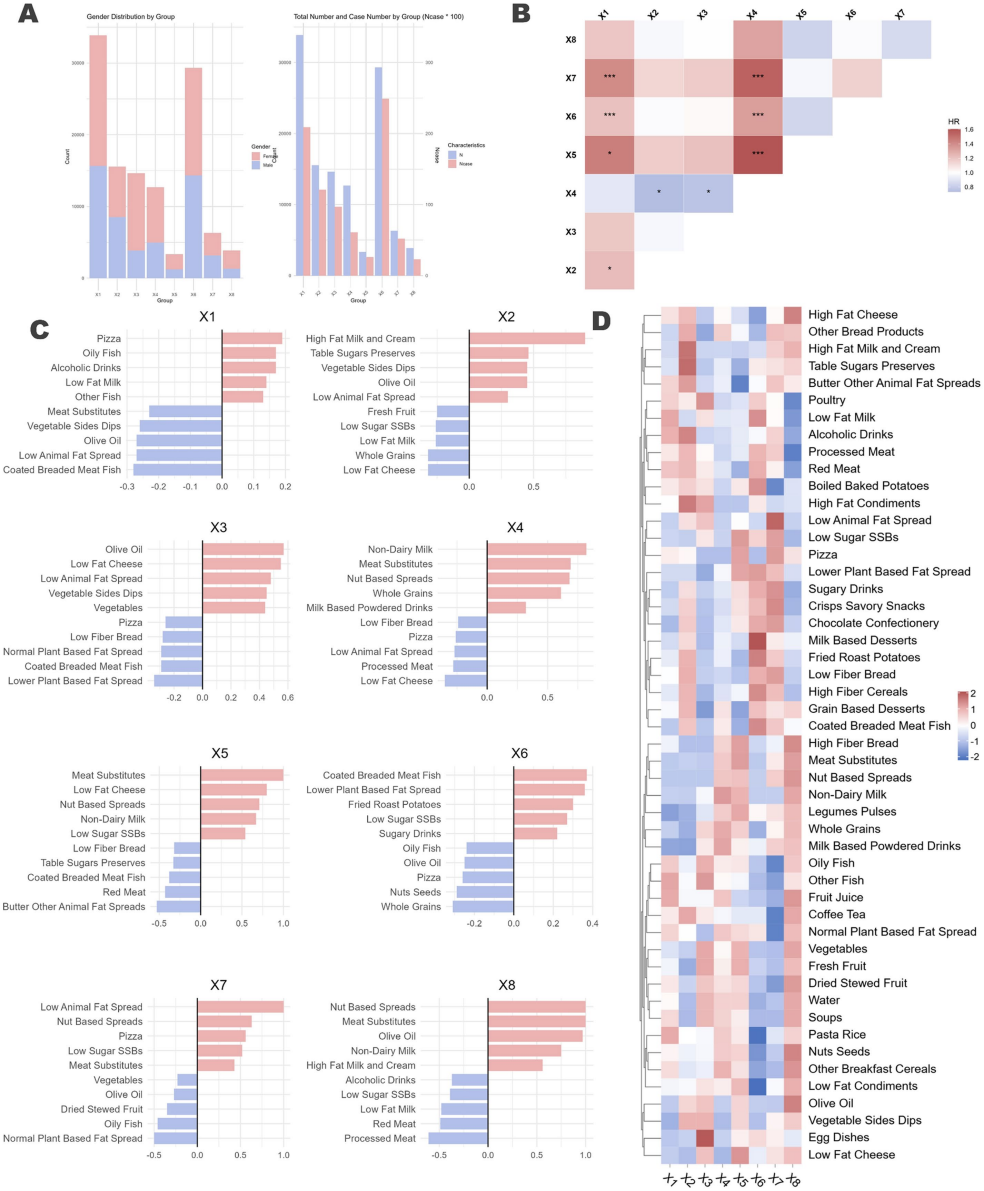
Latent profile analysis of naturally occurring dietary preferences in the population and the differential impact on ARC across profiles. **(A)** Proportion of different genders within each dietary profile, represented by a stacked bar chart, along with the number of individuals who developed ARC (x100) and the total number of individuals in each profile. **(B)** Each dietary profile was used as the reference group, with the remaining profiles treated as multi-categorical variables. Cox regression analysis was performed, adjusting for all previously mentioned confounding factors, to assess the association between dietary profiles and ARC. The horizontal axis represents the reference group. Significance levels were denoted as *p* < 0.1 by “*” and *p* < 0.05 by “***.” **(C)** Bidirectional bar charts depicting the top five and bottom five food groups for each dietary profile, highlighting the distinctive characteristics of each profile. Detailed descriptions can be found in the Supplementary material. **(D)** Loadings of the 50 food groups across each dietary profile.

An integration of three methods indicates that the optimal model is composed of eight latent profiles. Figure 5 illustrates the preferences of participants within each profile across the 50 food groups. Detailed baseline characteristics and the intake of key food groups for each profile are provided in Supplementary Table S27.

The analysis identified distinct DPs within the population. The gender ratio differences among the eight identified profiles were minimal (Figure 5A). Figure 5C displays the top and bottom five food groups with the highest and lowest factor loadings for each profile, respectively, while Figure 5D presents a heatmap showing the loadings of 50 food groups across the eight profiles.

Profile X1 is characterized by a high intake of high-fat dairy products and animal protein, including high-fat cream, red meat, and processed meat, indicating elevated consumption of saturated fats and protein. Profile X2 emphasizes healthy fats, with substantial consumption of olive oil, nuts, seeds, and low-fat dairy, highlighting a focus on healthy fat intake. Profile X3 is indicative of a diet rich in whole plant foods such as vegetables, legumes, and pulses, marked by low-fat and high-fiber content. Profile X4 consists predominantly of plant-based foods and substitutes, including meat substitutes, vegetable sides, and dips, suggesting a plant-based diet supplemented with processed substitutes. Profile X5 is characterized by high sugar intake, featuring sugary drinks, high-sugar desserts, and low-fat dairy. Profile X6 includes both alcohol and sugar, with high consumption of alcoholic beverages, low-sugar sugar-sweetened beverages (SSBs), dairy, and meat substitutes, resulting in elevated sugar and alcohol intake. Profile X7 is defined by high-fat condiments and vegetables, incorporating high-fat condiments, dairy products, vegetable sides, and dips, indicating high fat consumption with an emphasis on vegetables. Finally, Profile X8 represents a balanced and diverse diet, encompassing whole grains, fish, olive oil, and fresh fruit, emphasizing the intake of healthy fats and high-fiber foods.

The incidence of ARC varied across the different profiles (X1, 0.62%; X2, 0.78%; X3, 0.66%; X4, 0.48%; X5, 0.75%; X6, 0.85%; X7, 0.82%; X8, 0.59%; *χ*^2^ *p* = 0.001). We included the eight profiles as a categorical variable in the model, using each of the eight profiles sequentially as reference groups to conduct Cox regression analysis controlling for all confounders (Figure 5B). The results indicated significant associations for Profile X5 [1.604 (1.013, 2.540), *p* = 0.044], Profile X6 [1.342 (1.012, 1.781), *p* = 0.041], and Profile X7 [1.555 (1.071, 2.258), *p* = 0.020] when compared to Profile X4, which is representative of a plant-based diet. In summary, compared to Profile X4, Profiles X5, X6, and X7, which have higher intakes of sugar and high-fat foods, were associated with an increased risk of ARC.

## Discussion

In this large British cohort, we first examined the relationship between individual food groups, nutrients, metabolites, and genetic factors with ARC. We then developed a CDPI framework using RRR and LPA. While RRR allowed us to analyze DPs by linking key nutrients with disease pathways, LPA helped identify natural dietary subgroups in the population, shedding light on their associations with ARC risk.

The high-fat, calorie-dense diet and the hyperglycemic, fiber-deficient diet were constructed through RRR from six DPs across five nutrient combinations and four DPs from three nutrient combinations, respectively. These patterns consistently demonstrated significant impacts on ARC risk across different causal pathways. The high-fat, calorie-dense diet involved frequent consumption of high-fat cheese, red meat, and butter, alongside reduced intake of vegetables and fruits. Similarly, the hyperglycemic, fiber-deficient pattern was marked by a high intake of sugary foods, chocolate, and desserts, with a notable deficiency in fiber-rich foods. LPA results further supported these findings, showing that individuals adhering to these DPs had a 1.3 to 1.6 times greater risk of ARC compared to those following healthier, plant-based diets.

It is noteworthy that within the hyperglycemic dietary habit, the DP from G5.DP1 did not exhibit any association with ARC, despite having an explanatory power of 51.33% for the response variables and explaining more than 80% of total sugar intake. However, it only accounted for approximately 7% of energy density. This DP is characterized by a preference for fresh fruits, fruit juices, and other fructose-rich foods with fiber content, and relatively low intake of high-fat cheese. This may suggest that energy density remains a crucial factor in determining the dietary promotion of ARC. Nevertheless, this DP also showed no protective effect against ARC.

Mediation analysis identified several key metabolites, including urea, cystatin C, leucine, isoleucine, IGF-1, as significant mediators linking DPs to ARC risk. Elevated levels of urea and cystatin C, markers of early renal dysfunction, highlight that impaired kidney function can serve as an early indicator of cyst formation, particularly in response to unhealthy DPs (41, 42). Branched-chain amino acids, leucine and isoleucine, are potent activators of the mTORC1 pathway. These amino acids interact with cytosolic and lysosomal sensors, recruiting the mTOR complex to the lysosome, which subsequently activates downstream effectors such as p70S6K and 4E-BP1, promoting protein synthesis and cellular growth (43). This pathway plays a critical role in both normal growth and in pathological conditions like ARC, where aberrant activation of mTORC1 can lead to excessive cellular proliferation and impaired apoptosis, contributing to cyst expansion (44). Similarly, IGF-1, a potent growth factor that regulates cell proliferation and survival, activates mTORC1 via the PI3K/Akt signaling pathway (45). Chronic activation of mTORC1, particularly driven by elevated BCAAs or IGF-1 levels in response to high-fat, high-sugar diets, is a well-established mechanism underlying abnormal cell growth and organ hypertrophy (45–47). This dysregulation may represent a key driver in the progression of renal cysts. Kipp et al. (15) and Warner et al. (48) have demonstrated that caloric restriction can significantly inhibit cyst growth and slow disease progression by suppressing mTOR signaling, highlighting the therapeutic potential of dietary interventions to modulate this pathway. Their findings suggest that excessive mTORC1 activation—whether via nutrient overload, elevated BCAAs, or IGF-1 can exacerbate metabolic stress, promoting cyst growth and kidney dysfunction.

In the analysis of the relationship between three relatively healthy dietary habits derived from 17 DPs and ARC, the results did not show complete consistency. Among the micronutrient-rich and fiber-rich dietary habits, G4.DP1 and G4.DP2 were derived from the same set of nutrient response variables (vitamin C, vitamin E, beta-carotene, selenium). However, G4.DP1 showed a weak negative correlation with ARC, while G4.DP2 displayed no significant correlation but had a positive effect direction. G4.DP1 had good explanatory power for all four response variables, whereas G4.DP2 lacked explanatory power for vitamin C and beta-carotene, which was reflected in a poorer preference for vegetables and fruits in its food group. Another pattern, G6.DP1, originated from response variables related to iron metabolism and ultimately reflected high intake of vitamin C and vegetables/fruits. The G9.DP1 pattern, characterized by lean proteins and high fiber, demonstrated a strong protective effect against ARC, which is a novel finding. These results were further supported by regression analyses of individual food groups. Unfortunately, the mineral-rich diet did not show a significant correlation with ARC in the overall population. However, it is important to note that categorizing this type of diet broadly might be oversimplifying. Interestingly, in subgroup analyses by age, specifically among individuals aged 50–60, the three DPs under this dietary habit consistently showed a strong protective effect.

Existing studies have found that acidic urine is associated with impaired renal function and a higher prevalence of kidney cysts. In patients with a urine pH ≤5.0, the incidence of kidney cysts significantly increases (49, 50). Diets rich in vegetables and fruits, which are alkaline, can effectively reduce the renal acid load, potentially explaining their protective effect against ARC (51). This aligns with evidence-based nutritional care for patients with chronic kidney disease.

Our findings suggest that high-fiber and nutrient-rich diets have a protective effect against ARC, with hemoglobin concentration and red blood cell distribution width (RDW) potentially playing mediating roles. This relationship can be explained through the interaction of inflammation and oxidative stress, both of which are well-established contributors to cyst formation and progression. Chronic inflammation and oxidative stress induce damage to renal tubular epithelial cells, promoting cyst growth and fibrosis (52). DPs rich in fiber and minerals, known to reduce systemic inflammation, may mitigate these harmful processes by regulating gut microbiota and reducing pro-inflammatory cytokine production (52). Moreover, hemoglobin and RDW are markers of systemic health. Lower hemoglobin levels and elevated RDW have been associated with increased oxidative stress and chronic inflammation, which are risk factors for both kidney dysfunction and cyst development. Studies show that reducing RDW through dietary intervention could reduce the risk of kidney function decline, suggesting that modulating these blood markers through diet might explain part of the protective mechanism of high-fiber diets (53).

The strength of this study lies in the large UKB cohort. Additionally, controlling for the PRS of ARC allows us to minimize the confounding influence of genetic factors. The mediation analysis using metabolomics data was particularly beneficial in exploring the potential mechanisms by which DPs influence ARC risk. Lastly, the CDPI framework, constructed through a combination of RRR and LPA, provides a more comprehensive and systematic reflection of participants’ DPs, further enhancing the robustness of the analysis.

This study has several limitations. Firstly, identifying and validating DPs from at least two 24-h online self-reported dietary assessments may be subject to recall bias or misreporting. For instance, individuals with poorer overall health may report their dietary intake differently. Additionally, the identification of DPs relies on data from participants who are more willing to report their dietary intake, potentially introducing selection bias. We attempted to mitigate the randomness and partial bias of DPs by analyzing dietary habits derived from DPs across multiple nutrient combinations. However, not all foods are captured by the questionnaire, which introduces unknown biases. Secondly, there is a potential delay in identifying ARC cases (i.e., kidney cysts may not be immediately recognized upon occurrence). Furthermore, due to the nature of UKB data, we could not distinguish between simple and complex kidney cysts, nor could we account for cysts associated with end-stage renal disease. Selection related to repeated dietary assessments. The requirement of ≥2 valid WebQ assessments likely enriched the analytic cohort for individuals with greater digital access, health consciousness, and socio-economic advantage. Despite multivariable adjustment (education, deprivation, lifestyle), residual selection bias may remain. Because such individuals tend to report healthier diets and behaviors, between-person exposure contrast is reduced, which could bias harmful-diet associations toward the null (i.e., underestimation). Thirdly, DPs cannot explain all the variability of nutrient response variables included in the RRR model, and any residual variability might be attributable to other nutrients potentially involved in disease pathways. Fourthly, while LPA can uncover naturally occurring DPs within the population, the complexity of human diets and the limited data resulted in up to eight profiles in the best-fitting model. This complexity poses challenges in identifying the unique dietary characteristics of each profile and complicates the interpretation of differences in ARC risk among profiles. Lastly, it is important to note that the UKB is a non-probability sample (i.e., participants must respond to invitations to be included, and the sample is not entirely random). Most participants are White British and have a lower socioeconomic deprivation level than the UK average. Therefore, our findings may be conservative and not fully generalizable.

Our study found that lipid-rich, calorically dense diets (high-fat cheese, butter, pizza) and high-sugar, fiber-deficient diets (chocolate confectionery, sugary drinks) significantly increased ARC risk, while micronutrient-rich, low-lipid diets (vegetables, fresh fruit) and fiber-enriched diets (lean poultry, nuts, eggs) reduced it. Mineral-rich, moderate-fat diets showed no association. Branched-chain amino acids, IGF-1, and RBC distribution width played significant mediating roles in these dietary patterns’ associations with ARC risk.

## Data Availability

This study was conducted using data from the UK Biobank, and access to the dataset is restricted to approved researchers under institutional and ethical guidelines. Requests to access these datasets should be directed to 2291805677@qq.com.
