# Supplementary material for: Comprehensive dietary patterns explain acquired cystic kidney disease risk through genetic and metabolomic mechanisms
Source: Front Nutr. 2025 Oct 29;12:1611656. doi: 10.3389/fnut.2025.1611656 (PMC12605427; doi:10.3389/fnut.2025.1611656)
Supplement: Supplementary file 1 [file Data_Sheet_1.pdf]

Comprehensive Dietary Patterns Explain Acquired Cystic Kidney Disease Risk through Genetic and Metabolomic Mechanisms

Supplementary Materials

Supplementary Table S1: Food Group Classification Based on Nutritional Composition and Culinary Use.

Supplementary Note 1: The Schofield Equation for Estimating Energy Requirements.

Supplementary Figure S1: Theoretical direct acyclic graph guiding the analyses.

Supplementary Figure S2: Flow Chart of Study.

Supplementary Table S2: Categorization of Health Focus Areas and Corresponding Major Nutrients (Response Variables).

Supplementary Table S3 - S11: Average and Cumulative Explained Variation in Food Intake and Nutrient Response Variables for Each Dietary Pattern in Group 1 to Group 9.

Supplementary Table S12: HRs (95% CIs) for ARC by Each Food Group.

Supplementary Table S13: HRs (95% CIs) for ARC by Each Nutrients.

Supplementary Table S14: Key Food Groups with Highest and Lowest Factor Loadings in Dietary Patterns with Average Explained Variation Greater Than 15% for Each Health Focus Response Variable Combination (N=119,709).

Supplementary Figure S3 – S7: Factor Loadings for Food Groups in Dietary Patterns for Each Health Focus Response Variable Combination with Participants Completing at Least Two 24-Hour Dietary Questionnaires.

Supplementary Table S15: Quartile Stratification of Dietary Patterns within Nutrient Groups Based on Z-Scores with Regression Outcomes Referenced to Quartile 1.

Supplementary Table S16 – S26: Stratified Analysis of Dietary Patterns for Each Health Focus Response Variable Combination and Their Association with ARC Based on Gender, Age, Townsend deprivation index, Education score, BMI, Smoking status, Alcohol status, Physical activity level (IPAQ), Diabetes, Cardiovascular disease and hypertension.

Supplementary Table S27: Baseline characteristics of participants by latent profile (N = 119,709).

Supplementary Table S28: Variables and medication codes used to define MetS in the UKB cohort.

Supplementary Table S29: Detailed description of UKB medication codes (mapped to ATC codes) used to define MetS components

Supplementary Figure S7:

eTable1: Baseline characteristics of participants by dietary patterns (N = 119,709)

eTable2: Associations of Metabolomics Biomarkers with 50 Food Groups and 63 Nutrients After Controlling for Confounding Variables

eTable3: Associations of Metabolomics Biomarkers with 17 Ditery Patterns After Controlling for Confounding Variables

(The results of the above 3 eTables are presented in the supplementary Excel file.)

Supplementary Table S1

**Food group**	**Food items**

High-fat milk and cream	Whole milk >3.6 g fat per 100 g
	Cream
	Full fat yogurt
Low-fat milk	Semi skimmed milk >1 g fat per 100 g
	Skimmed milk
	Low fat yogurt
Non-dairy milk	Rice/oat milk
	Soya
High fat cheese	High fat cheese
Low fat cheese	Low fat cheese
Olive oil	Olive oil (drizzling/dunking)
Poultry	Poultry
Red meat	Pork
	Beef
	Lamb
	Other meat
Processed meat	Processed meat
Oily fish	Oily fish
Other fish	Shellfish
	White fish & tinned tuna
Coated or breaded meat and fish	Breaded/battered chicken
	Breaded/battered fish
Egg & egg dishes	Egg & egg dishes
Meat substitutes	Meat substitutes - vegetarian
	Meat substitutes - soy
Legumes & pulses	Legumes & pulses
Vegetables	Raw salad
	Green leafy/cabbages
	Root vegetables
	Tomatoes
	Allium vegetables
	Other vegetables, including mushrooms, fruiting and mixed veg
	Peas/sweetcorn
Boiled or baked potatoes	Potatoes/Sweet potatoes (baked/boiled)
	Mashed potatoes
Fried or roast potatoes	Fried/roast potatoes
Low-fiber bread	White bread
High-fiber bread	Wholemeal bread
	Mixed (50/50), brown & seeded
Other bread products	Other bread
	Savoury crackers
	Grain dishes - added fat
	Samosa, pakora

Fresh fruit	Citrus
	Berries
	Apples & pears
	Other fruit
Dried and stewed fruit	Dried fruit
	Stewed fruit
Pasta and rice	White pasta & rice
	Sushi
Wholemeal pasta, brown rice & other wholegrains	Wholemeal pasta, brown rice & other wholegrains
Pizza	Pizza
Nuts & seeds	Salted nuts & seeds
	Unsalted nuts & seeds
Fruit juice	Fruit juice
Coffee and tea	Coffee, caffeinated
	Coffee, decaffeinated
	Tea
	Tea, decaffeinated
Sugar-sweetened beverages & other sugary drinks	Sugar-sweetened beverages & other sugary drinks
Low/non sugar SSBs	Low/non sugar SSBs
Alcoholic drinks(Wine, beer, spirits)	White wine
	Red wine
	Fortified wine
	Beer & Cider
	Spirits
Water/Sparkling water	Water/Sparkling water
Milk-based and powdered drinks	Milk-based and powdered drinks
Soups	Soups
Sauces & condiments (high fat)	Sauces & condiments (high fat)
Sauces & condiments (low fat)	Sauces & condiments (low fat)
Milk-based desserts	Milk-dairy desserts
	Soy desserts and yogurt
Grain-based desserts	Other desserts & cakes & pastries
Table sugars & preserves	Table sugars & preserves
Chocolate and confectionery	Chocolate confectionary
	Other sweets
High-fiber breakfast cereals	Bran cereal
	Biscuit cereal
	Porridge
Other breakfast cereals	Oat cereal (sugar)
	Muesli
	Other cereal (sugar)

Crisps and savoury snacks	Savoury snacks
Vegetable side dishes and dips	Vegetable side dishes
	Vegetable dips
Low animal fat spread	Reduced fat animal fat spread
Butter and other animal fat spreads	Butter and other animal fat spreads
Lower plant-based fat spread	Reduced fat plant-based fat spread
Normal plant-based fat spread	Plant-based fat spread
Nut-based spread	Nut-based spread

Supplementary Table S1 | Food Group Classification Based on Nutritional Composition and Culinary Use. This table presents the classification of food intake data into 50 main groups. The classification aligns with the U.K. National Diet and Nutrition Survey and is based on the similarity of their nutritional composition and culinary use. The methodology used for this aggregation follows previously described methods, ensuring consistency and accuracy in the categorization process.

Supplementary Note 1: The Schofield Equation for Estimating Energy Requirements

1.The Schofield Equation:

For Males:

Age 10-18: BMR (kcal/day) = 17.686 × weight (kg) + 658.2

Age 18-30: BMR (kcal/day) = 15.057 × weight (kg) + 692.2

Age 30-60: BMR (kcal/day) = 11.472 × weight (kg) + 873.1

Age >60: BMR (kcal/day) = 11.711 × weight (kg) + 587.7

For Females:

Age 10-18: BMR (kcal/day) = 13.384 × weight (kg) + 692.6

Age 18-30: BMR (kcal/day) = 14.818 × weight (kg) + 486.6

Age 30-60: BMR (kcal/day) = 8.126 × weight (kg) + 845.6

Age >60: BMR (kcal/day) = 9.082 × weight (kg) + 658.5

2.Calculation of EER:

To estimate the EER, the calculated BMR is multiplied by a Physical Activity Level (PAL) factor, which accounts for the individual's level of physical activity. In our study, PAL was determined based on the International Physical Activity Questionnaire (IPAQ):

Low activity: PAL = 1.4

Moderate activity: PAL = 1.6

High activity: PAL = 1.8

3.Dietary Assessment:

Under-reporters: EI: EER < lower 95% CI of EI: EER

Over-reporters: EI: EER > upper 95% CI of EI: EER

Supplementary Figure S1

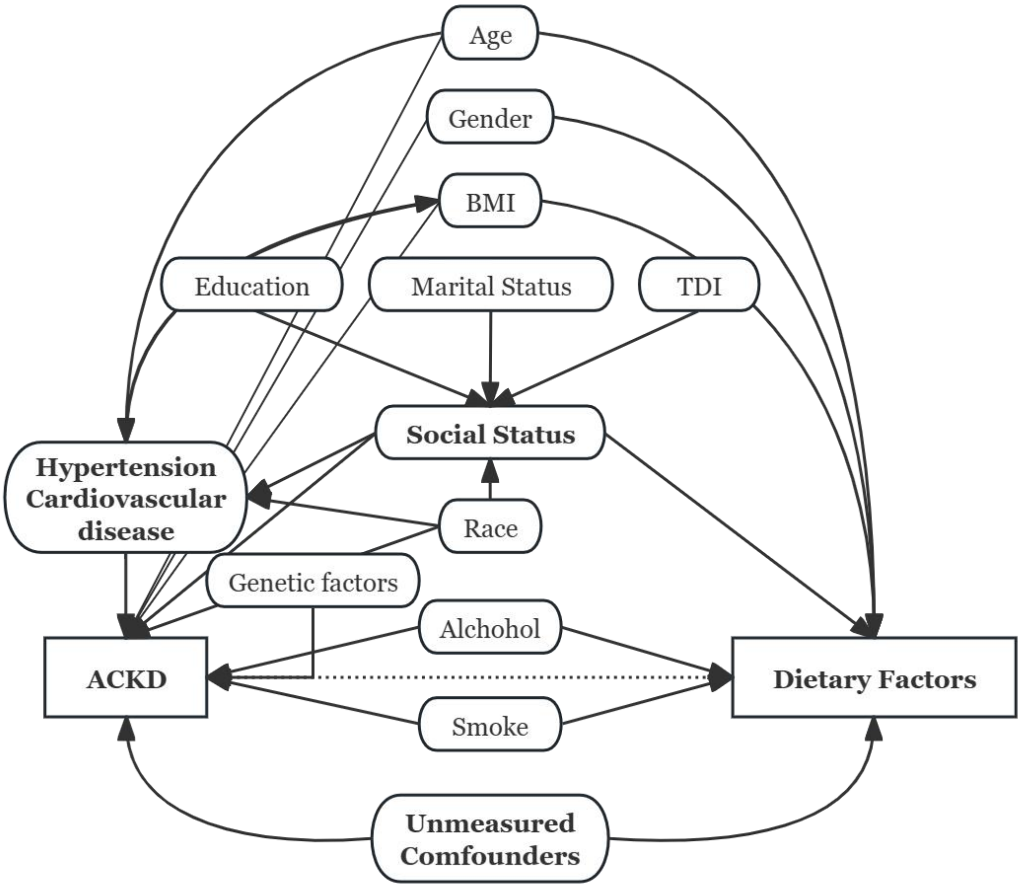

Supplementary Figure S1 | Theoretical direct acyclic graph guiding the analyses.

Supplementary Figure S2

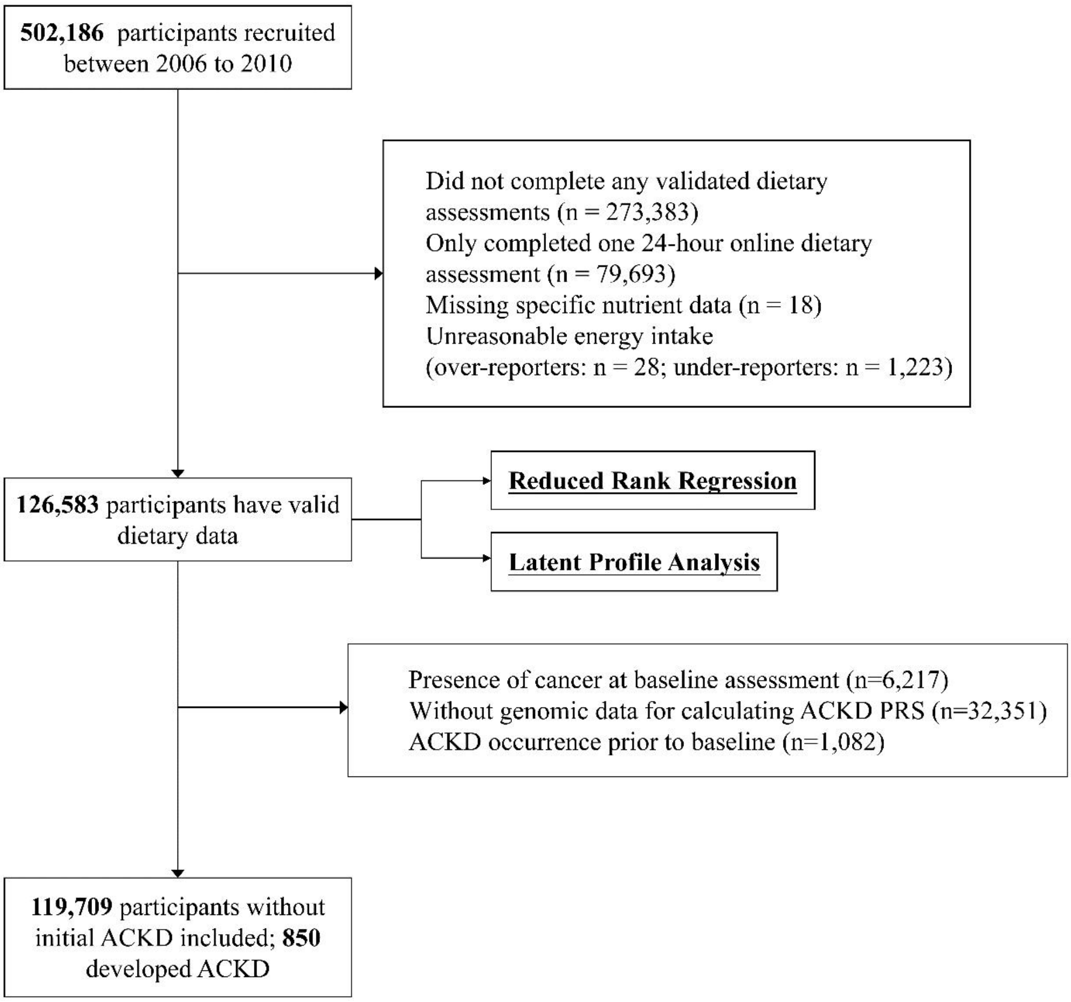

Supplementary Figure S2 | Flow Chart of Study

Supplementary Table S2

Group	Health Focus	Major Nutrients (Response Variables)
G1	Basic Nutrition and Energy Balance	Energy density, SFA, Sugar, Fibre
G2	Cardiovascular Health	Saturated fatty acids, Trans fatty acids, Cholesterol, Ω-3 fatty acids, Ω-6 fatty acids
G3	Bone Health	Vitamin D, Calcium, Magnesium, Phosphorus
G4	Antioxidation and Cellular Protection	Vitamin C, Vitamin E, Beta-carotene, Selenium
G5	Glycemic Control	Total sugars, Sucrose, Fructose, Fibre
G6	Iron Metabolism	Iron, Folate, VitaminB12, Vitamin C
G7	Blood Pressure Management	Sodium, Potassium, Calcium, Magnesium
G8	Renal Protection	Protein, Sodium, Potassium, Phosphorus
G9	Metabolic Health	Biotin, Niacin equivalent, Pantothenic acid

Supplementary Table S2 | Categorization of Health Focus Areas and Corresponding Major Nutrients (Response Variables).

Supplementary Table S3

No.	Energy density (kJ/g)	Saturated fat acids (%E)	Free sugars (%E)	Fiber density(g/MJ)	Average	Cumulative average
f1	67.95	29.18	36.65	55.08	**47.22**	47.22
f2	1.69	9.44	41.20	4.42	**15.69**	60.90
f3	2.77	30.40	2.20	11.40	11.69	72.60
f4	9.24	0.39	0.11	7.06	4.20	76.79

Supplementary Table S3 | Average and Cumulative Explained Variation in Food Intake and Nutrient Response Variables for Each DP in Group1 (N=119,709)

Supplementary Table S4

No.	Energy density (kJ/g)	Saturated fat acids (g/d)	Trans fatty acids (g/d)	Cholesterol (g/d)	Ω-3 fatty acids (g/d)	Ω-6 fatty acids (g/d)	Average	Cumulative average
f1	22.85	70.16	46.96	39.12	38.48	50.40	**44.66**	44.66
f2	37.16	6.12	6.58	6.60	27.07	7.24	**15.13**	59.79
f3	2.31	0.07	0.81	44.87	6.43	14.72	11.53	71.32
f4	17.80	5.18	14.33	5.52	0.03	1.62	7.41	78.74
f5	1.39	0.06	0.03	0.20	12.21	12.86	4.46	83.20
f6	0.14	3.81	2.89	0.01	0.05	0.02	1.15	84.35

Supplementary Table S4 | Average and Cumulative Explained Variation in Food Intake and Nutrient Response Variables for Each DP in Group2 (N=119,709)

Supplementary Table S5

No.	Energy density (kJ/g)	Vitamin D (g/d)	Calcium (g/d)	Magnesium (g/d)	Phosphorus (g/d)	Average	Cumulative average
f1	0.15	19.16	58.22	70.73	81.79	**46.01**	46.01
f2	78.00	2.54	1.99	1.88	0.23	**16.93**	62.94
f3	2.45	63.61	4.40	2.50	0.47	14.69	77.63
f4	1.01	0.24	13.64	10.99	0.05	5.19	82.82
f5	0.03	0.06	0.79	1.22	3.64	1.15	83.96

Supplementary Table S5 | Average and Cumulative Explained Variation in Food Intake and Nutrient Response Variables for Each DP in Group3 (N=119,709)

Supplementary Table S6

No.	Energy density (kJ/g)	Vitamin C (g/d)	Vitamin E (g/d)	Beta-carotene (g/d)	Selenium (g/d)	Average	Cumulative average
f1	30.82	61.41	32.47	37.38	23.51	**37.12**	37.12
f2	38.46	4.68	31.97	2.73	36.41	**22.85**	59.97
f3	7.97	3.17	12.70	1.33	27.96	10.63	70.59
f4	2.09	12.90	2.05	5.54	0.25	4.57	75.16
f5	2.31	0.03	1.95	4.57	0.16	1.80	76.97

Supplementary Table S6 | Average and Cumulative Explained Variation in Food Intake and Nutrient Response Variables for Each DP in Group4 (N=119,709)

Supplementary Table S7

No	Energy density (kJ/g)	Total sugars (g/d)	Sucrose (g/d)	Fructose (g/d)	Englyst fiber (g/d)	Average	Cumulative average
f1	7.26	81.04	50.10	65.50	52.74	**51.33**	51.33
f2	63.03	6.38	31.84	6.63	7.92	**23.16**	74.49
f3	8.21	1.18	2.31	0.65	22.99	7.07	81.56
f4	3.15	0.10	3.53	8.21	1.21	3.24	84.80
f5	0.00	1.36	0.57	0.37	0.00	0.46	85.26

Supplementary Table S7 | Average and Cumulative Explained Variation in Food Intake and Nutrient Response Variables for Each DP in Group5 (N=119,709)

Supplementary Table S8

No	Energy density (kJ/g)	Iron (g/d)	Folate (g/d)	Vitamin B12 (g/d)	Vitamin C (g/d)	Average	Cumulative average
f1	20.49	39.88	67.76	9.88	61.09	**39.82**	39.82
f2	51.11	22.50	3.08	12.11	8.64	**19.49**	59.31
f3	4.77	1.79	1.45	34.15	1.61	8.75	68.07
f4	5.19	3.46	0.58	0.94	10.46	4.13	72.19
f5	0.08	2.29	3.60	0.00	0.39	1.27	73.47

Supplementary Table S8 | Average and Cumulative Explained Variation in Food Intake and Nutrient Response Variables for Each DP in Group6 (N=119,709)

Supplementary Table S9

No.	Energy density (kJ/g)	Sodium (g/d)	Potassium (g/d)	Magnesium (g/d)	Average	Cumulative average
f1	0.00	44.24	71.78	74.63	**48.95**	48.95
f2	73.20	24.08	12.24	2.36	**22.80**	71.75
f3	0.85	1.82	2.14	2.52	6.01	77.76
f4	7.17	13.90	0.29	4.35	5.16	82.92
f5	0.43	0.07	4.26	3.47	1.65	84.57

Supplementary Table S9 | Average and Cumulative Explained Variation in Food Intake and Nutrient Response Variables for Each DP in Group7 (N=119,709)

Supplementary Table S10

No.	Energy density (kJ/g)	Protein (g/d)	Sodium (g/d)	Potassium (g/d)	Phosphorus (g/d)	Average	Cumulative average
f1	0.98	80.09	53.73	60.06	80.08	**54.99**	54.99
f2	74.32	0.12	14.46	21.07	0.19	**22.03**	77.02
f3	1.28	8.45	13.78	0.64	0.49	4.93	81.95
f4	4.95	4.42	2.08	7.65	0.41	3.90	85.85
f5	0.12	1.04	0.06	1.30	5.01	1.51	87.35

Supplementary Table S10 | Average and Cumulative Explained Variation in Food Intake and Nutrient Response Variables for Each DP in Group8 (N=119,709)

Supplementary Table S11

No.	Biotin (g/d)	Niacin equivalent (g/d)	Pantothenic acid (g/d)	Average	Cumulative average
f1	55.74	73.72	70.12	**66.53**	66.53
f2	21.57	8.94	1.16	10.55	77.08
f3	0.81	7.16	12.57	6.85	83.93

Supplementary Table S11 | Average and Cumulative Explained Variation in Food Intake and Nutrient Response Variables for Each DP in Group9 (N=119,709)

Supplementary Table S12

Food Groups	P value	HR
Normal Plant Based Fat Spread	0.327	0.994 [0.982, 1.006]
Whole Grains	0.103	0.997 [0.994, 1.001]
Low Fat Condiments	0.427	0.998 [0.993, 1.003]
Nuts Seeds	0.550	0.998 [0.993, 1.004]
Dried Stewed Fruit	0.120	0.998 [0.996, 1.0001]
**Poultry**	**0.042**	**0.998 [0.996, 1.0001]**
Coated Breaded Meat Fish	0.573	0.999 [0.996, 1.002]
Other Fish	0.518	0.999 [0.996, 1.002]
Pizza	0.515	0.999 [0.997, 1.001]
Non-Dairy Milk	0.512	0.999 [0.997, 1.001]
Egg Dishes	0.330	0.999 [0.997, 1.001]
Other Breakfast Cereals	0.711	0.999 [0.997, 1.002]
Soups	0.084	0.999 [0.998, 1.0001]
Vegetable Sides Dips	0.999	1.0001 [0.995, 1.005]
Meat Substitutes	0.993	1.0001 [0.996, 1.004]
Oily Fish	0.976	1.0001 [0.997, 1.003]
Processed Meat	0.971	1.0001 [0.997, 1.003]
Legumes Pulses	0.916	1.0001 [0.997, 1.003]
Fried Roast Potatoes	0.507	1.0001 [0.998, 1.001]
Low Fat Milk	0.613	1.0001 [0.999, 1.0001]
Vegetables	0.197	1.0001 [0.999, 1.0001]
**Fresh Fruit**	**0.046**	**0.9999 [0.999, 1.0000]**
**Coffee Tea**	**0.003**	**0.9999 [0.999, 1.0000]**
Fruit Juice	0.937	1.0001 [0.999, 1.001]
Milk Based Powdered Drinks	0.784	1.0001 [0.999, 1.001]
Pasta Rice	0.751	1.0001 [0.999, 1.001]
High Fat Milk and Cream	0.709	1.0001 [0.999, 1.001]
Low Sugar SSBs	0.600	1.0001 [1.0001, 1.0001]
Water	0.527	1.0001 [1.0001, 1.0001]
Alcoholic Drinks	0.074	1.0001 [1.0001, 1.0001]
High Fiber Cereals	0.549	1.001 [0.998, 1.005]
Red Meat	0.471	1.001 [0.999, 1.002]
Milk Based Desserts	0.364	1.001 [0.999, 1.002]
Other Bread Products	0.491	1.001 [0.999, 1.003]
High Fiber Bread	0.285	1.001 [0.999, 1.003]
Low Fiber Bread	0.275	1.001 [0.999, 1.003]
Grain Based Desserts	0.199	1.001 [0.999, 1.003]
**Sugary Drinks**	**0.001**	**1.001 [1.0001, 1.001]**
Boiled Baked Potatoes	0.168	1.001 [1.0001, 1.002]
Lower Plant Based Fat Spread	0.692	1.002 [0.990, 1.015]
Chocolate Confectionery	0.169	1.002 [0.999, 1.006]
Nut Based Spreads	0.884	1.003 [0.964, 1.044]

Crisps Savory Snacks	0.206	1.003 [0.998, 1.008]
High Fat Condiments	0.053	1.004 [1.0001, 1.009]
Butter Other Animal Fat Spreads	0.171	1.005 [0.998, 1.013]
Low Fat Cheese	0.099	1.006 [0.999, 1.013]
**High Fat Cheese**	**0.002**	**1.006 [1.002, 1.010]**
**Table Sugars Preserves**	**0.008**	**1.006 [1.002, 1.011]**
Low Animal Fat Spread	0.216	1.011 [0.994, 1.029]
Olive Oil	0.350	1.028 [0.970, 1.089]

Supplementary Table S12 | HRs (95% CIs) for ARC by Each Food Group.

All models adjusted for demographic and socio-economic covariates, including gender, age at recruitment, socio-economic status (Townsend deprivation index), employment status, educational attainment, health status, and log-transformed total caloric intake, lifestyle and health conditions, including average hours of sleep per night, current smoking status, Body Mass Index (BMI), current alcohol consumption status, physical activity level (IPAQ), Polygenic Risk Score (PRS), heart disease, hypertension, and diabetes.

Supplementary Table S13

Nutrients	P value	HR
**Vitamin B6**	**0.018**	**0.843 [0.733, 0.971]**
Copper	0.174	0.867 [0.706, 1.065]
Riboflavin	0.137	0.891 [0.766, 1.037]
Fiber New	0.079	0.898 [0.796, 1.013]
Thiamin	0.197	0.915 [0.799, 1.047]
**Pantothenic Acid**	**0.017**	**0.943 [0.899, 0.990]**
Manganese	0.102	0.950 [0.894, 1.010]
n-3 Fatty Acids	0.490	0.967 [0.879, 1.064]
Non-Haem Iron	0.064	0.971 [0.940, 1.002]
Iron	0.060	0.971 [0.941, 1.001]
Haem Iron	0.748	0.973 [0.822, 1.151]
Total Nitrogen	0.386	0.986 [0.956, 1.018]
Vitamin B12	0.310	0.988 [0.965, 1.011]
n-6 Fatty Acids	0.269	0.988 [0.968, 1.009]
Englyst Fiber	0.080	0.988 [0.974, 1.002]
**Niacin Equivalent**	**0.016**	**0.988 [0.979, 0.998]**
Vitamin E	0.379	0.989 [0.966, 1.013]
Zinc	0.580	0.991 [0.958, 1.024]
**Biotin**	**0.005**	**0.991 [0.986, 0.997]**
Vegetable Protein	0.229	0.994 [0.984, 1.004]
Vitamin D	0.740	0.995 [0.967, 1.024]
Maltose	0.392	0.995 [0.982, 1.007]
**Alcohol**	**0.040**	**0.996 [0.993, 1.000]**
Lactose	0.561	0.997 [0.987, 1.007]
Vegetable Fat	0.348	0.997 [0.991, 1.003]
Protein	0.185	0.997 [0.992, 1.002]
Selenium	0.110	0.997 [0.993, 1.001]
Intrinsic and Milk Sugars	0.091	0.997 [0.994, 1.000]
Animal Protein	0.475	0.998 [0.994, 1.003]
**Magnesium**	**0.002**	**0.998 [0.997, 0.999]**
Fructose	0.653	0.999 [0.993, 1.004]
Glucose	0.792	0.999 [0.993, 1.006]
Folate	0.089	0.999 [0.998, 1.000]
Iodine	0.062	0.999 [0.998, 1.000]
Vitamin C	0.160	0.999 [0.998, 1.000]
Cholesterol	0.413	1.000 [0.999, 1.000]
Phosphorus	0.086	1.000 [0.999, 1.000]
Alpha Carotene	0.436	1.000 [1.000, 1.000]
Beta Carotene	0.187	1.000 [1.000, 1.000]
Beta Cryptoxanthin	0.386	1.000 [1.000, 1.000]
Calcium	0.988	1.000 [1.000, 1.000]
Chloride	0.542	1.000 [1.000, 1.000]

Energy from Beverages	0.660	1.000 [1.000, 1.000]
**Potassium**	**0.005**	**1.000 [1.000, 1.000]**
Retinol	0.716	1.000 [1.000, 1.000]
Sodium	0.458	1.000 [1.000, 1.000]
Total Carotene	0.204	1.000 [1.000, 1.000]
**Total Weight of All Foods and Beverages**	**<0.001**	**1.000 [1.000, 1.000]**
**Total Weight of Beverages Only**	**0.006**	**1.000 [1.000, 1.000]**
Vitamin A Retinol Equivalents	0.819	1.000 [1.000, 1.000]
Nutrition Score	0.464	1.000 [1.000, 1.000]
Energy New	0.448	1.000 [1.000, 1.000]
Starch	0.332	1.001 [0.999, 1.003]
Total Sugars	0.211	1.001 [0.999, 1.003]
Carbohydrate	0.074	1.002 [1.000, 1.003]
Monounsaturated Fatty Acids	0.571	1.003 [0.992, 1.015]
Fat	0.180	1.003 [0.999, 1.008]
**Non-Milk Extrinsic Sugars**	**0.002**	**1.004 [1.001, 1.006]**
**Free Sugar**	**<0.001**	**1.004 [1.002, 1.007]**
**Sucrose**	**0.003**	**1.005 [1.002, 1.008]**
**Animal Fat**	**0.027**	**1.006 [1.001, 1.011]**
**Saturated Fatty Acids**	**0.009**	**1.012 [1.003, 1.021]**
Other Sugars	0.298	1.014 [0.988, 1.041]
**Energy Density**	**0.005**	**1.071 [1.022, 1.123]**
Trans Fatty Acids	0.091	1.124 [0.981, 1.286]

Supplementary Table S13 | HRs (95% CIs) for ARC by Each Nutrients.

All models adjusted for demographic and socio-economic covariates, including gender, age at recruitment, socio-economic status (Townsend deprivation index), employment status, educational attainment, health status, and log-transformed total caloric intake, lifestyle and health conditions, including average hours of sleep per night, current smoking status, Body Mass Index (BMI), current alcohol consumption status, physical activity level (IPAQ), Polygenic Risk Score (PRS), heart disease, hypertension, and diabetes.

Supplementary Table S14

	Explained variation	the Highest Loadings	the Lowest Loadings
**G1**	**Energy density, SFA, Sugar, Fibre**		
DP1	47.21%	Butter Other Animal Fat Spreads<br>High Fat Cheese<br>Red Meat<br>Grain Based Desserts	Vegetables<br>Fresh Fruit
DP2	15.69%	Chocolate Confectionery<br>Sugary Drinks<br>Table Sugars Preserves	Vegetables<br>Boiled Baked Potatoes<br>Fried Roast Potatoes<br>Pasta Rice
**G2**	**Energy density, Saturated fatty acids, Trans fatty acids, Cholesterol, Ω-3 fatty acids, Ω-6 fatty acids**		
DP1	44.67%	Egg Dishes<br>Butter Other Animal Fat Spreads<br>Red Meat<br>High Fat Cheese<br>Grain Based Desserts	Fresh Fruit
DP2	15.13%	High Fat Cheese<br>Chocolate Confectionery<br>Butter Other Animal Fat Spreads<br>High Fiber Cereals	Egg Dishes<br>Vegetables<br>Oily Fish
**G3**	**Energy density, Vitamin D, Calcium, Magnesium, Phosphorus**		
DP1	46.01%	Low Fat Milk<br>Oily Fish<br>High Fat Cheese<br>Pizza	Olive Oil<br>Table Sugars Preserves<br>Vegetable Sides Dips
DP2	16.93%	Pizza<br>Butter Other Animal Fat Spreads<br>High Fat Cheese<br>Chocolate Confectionery	Fresh Fruit<br>Vegetables<br>Soups<br>Boiled Baked Potatoes
**G4**	**Energy density, Vitamin C, Vitamin E, Beta-carotene, Selenium**		
DP1	37.12%	Vegetables<br>Fresh Fruit<br>Fruit Juice<br>Other Fish	Alcoholic Drinks<br>Chocolate Confectionery<br>High Fat Cheese
DP2	22.84%	Other Fish<br>Oily Fish<br>Egg Dishes<br>Nuts Seeds	Fresh Fruit<br>Vegetables
**G5**	**Energy density, Total sugars, Sucrose, Fructose, Fibre**		

DP1	51.33%	Fresh Fruit<br>Fruit Juice<br>Sugary Drinks<br>Table Sugars Preserves<br>Vegetables	Red Meat<br>High Fat Cheese<br>Butter Other Animal Fat Spreads
DP2	23.16%	Chocolate Confectionery<br>Table Sugars Preserves<br>Grain Based Desserts<br>Sugary Drinks	Vegetables<br>Fresh Fruit<br>Soups<br>Boiled Baked Potatoes
**G6**	**Energy density, Iron, Folate, Vitamin B12, Vitamin C**		
DP1	39.82%	Vegetables<br>Fresh Fruit<br>Fruit Juice<br>Low Fat Milk	Chocolate Confectionery<br>Table Sugars Preserves
DP2	19.49%	Chocolate Confectionery<br>Table Sugars Preserves<br>Olive Oil<br>Crisps Savoury Snacks	Fresh Fruit<br>Vegetables<br>Fruit Juice<br>Soups<br>Boiled Baked Potatoes
**G7**	**Energy density, Sodium, Potassium, Calcium, Magnesium**		
DP1	48.95%	Low Fat Milk<br>Pizza<br>Vegetables<br>Fresh Fruit<br>Alcoholic Drinks	Olive Oil<br>Table Sugars Preserves<br>Low Sugar SSBs
DP2	22.80%	Processed Meat<br>Pizza<br>High Fat Cheese<br>Butter Other Animal Fat Spreads	Fresh Fruit<br>Vegetables<br>Boiled Baked Potatoes<br>Dried Stewed Fruit
**G8**	**Energy density, Protein, Sodium, Potassium, Phosphorus**		
DP1	54.99%	Red Meat<br>Poultry<br>Low Fat Milk<br>Processed Meat<br>Pizza	Olive Oil<br>Table Sugars Preserves
DP2	22.03%	Butter Other Animal Fat Spreads<br>Processed Meat<br>Pizza<br>High Fat Cheese	Fresh Fruit<br>Vegetables<br>Boiled Baked Potatoes<br>Low Fat Milk<br>Dried Stewed Fruit
**G9**	**Biotin, Niacin equivalent, Pantothenic acid**		

DP1	66.53%	Poultry<br>Low Fat Milk<br>Red Meat<br>Nuts Seeds<br>Egg Dishes	Olive Oil<br>Low Sugar SSBs

Supplementary Table S14 | Key Food Groups with Highest and Lowest Factor Loadings in Dietary Patterns with Average Explained Variation Greater Than 15% for Each Health Focus Response Variable Combination (N=119,709).

Supplementary Figure S3

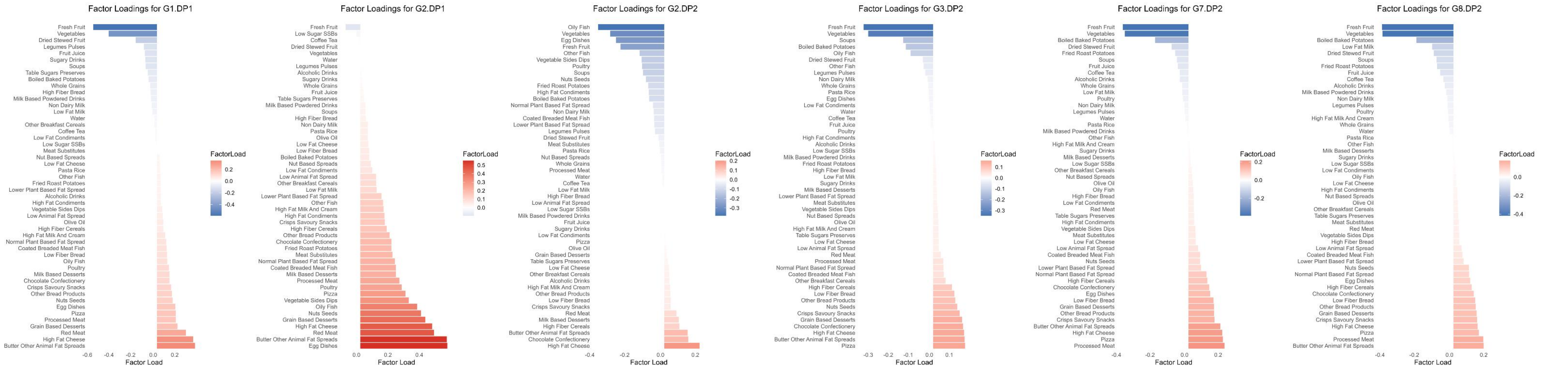

Supplementary Figure S3 | Factor Loadings for Food Groups in Dietary Patterns for Lipid-Rich, Calorically Dense Diet

Supplementary Figure S4

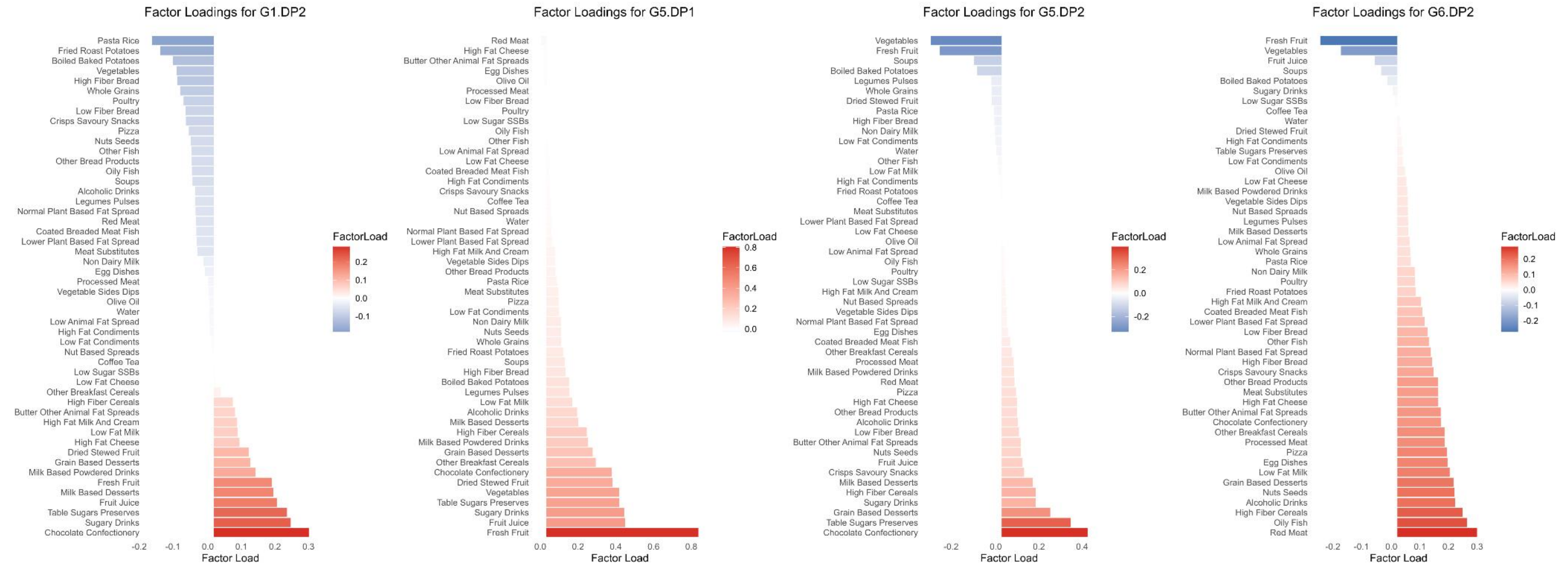

Supplementary Figure S4 | Factor Loadings for Food Groups in Dietary Patterns for Hyperglycemic, Fiber-Deficient Diet

Supplementary Figure S5

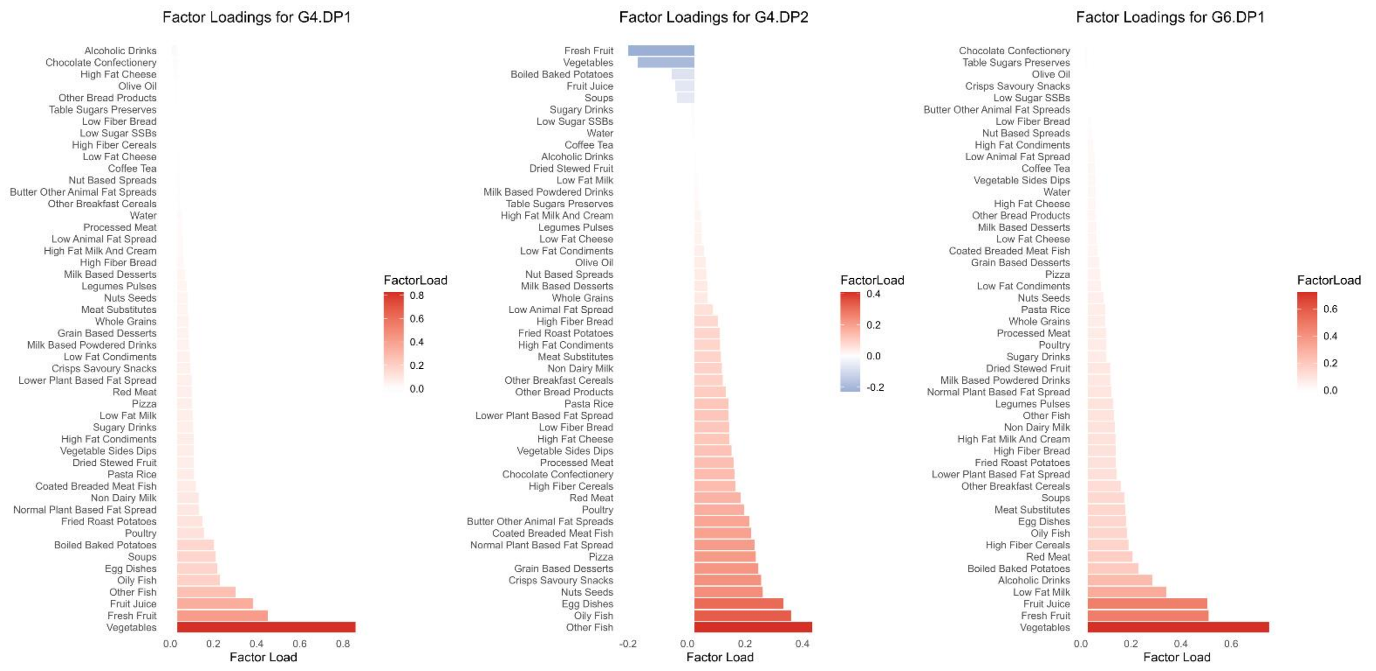

Supplementary Figure S5 | Factor Loadings for Food Groups in Dietary Patterns for Micronutrient-Abundant, Low-Lipid Diet

Supplementary Figure S6

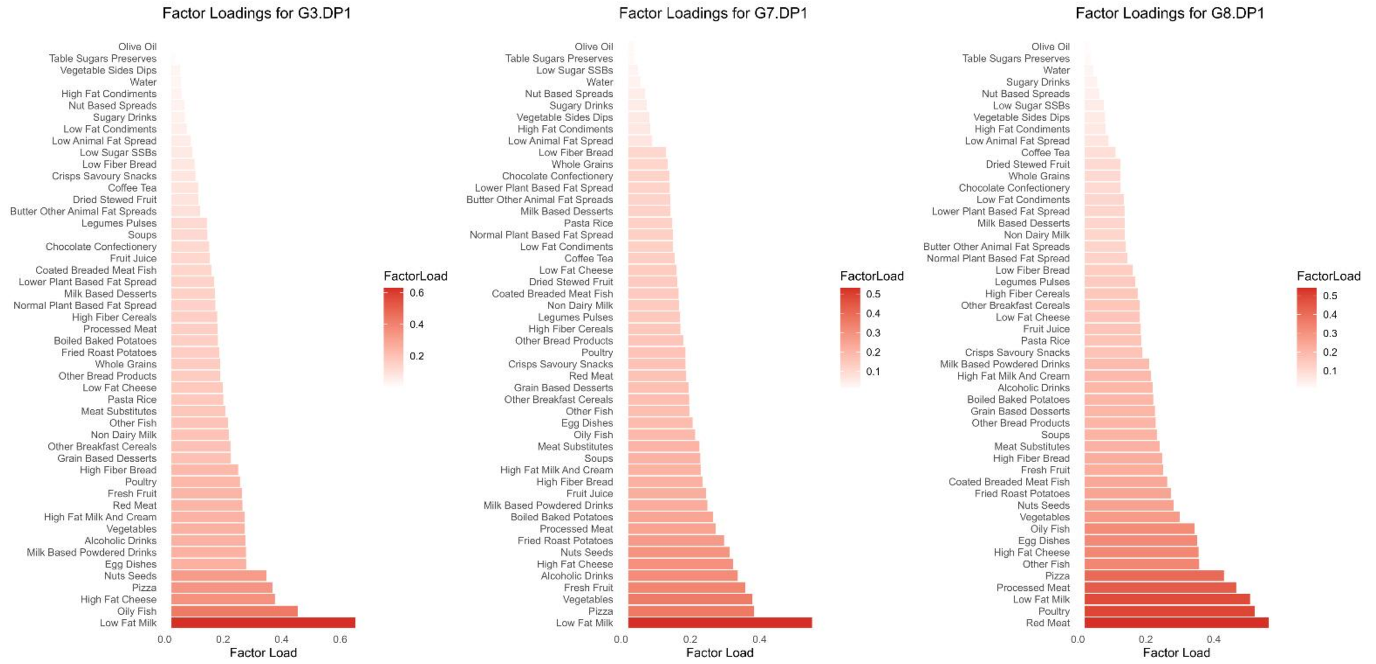

Supplementary Figure S7

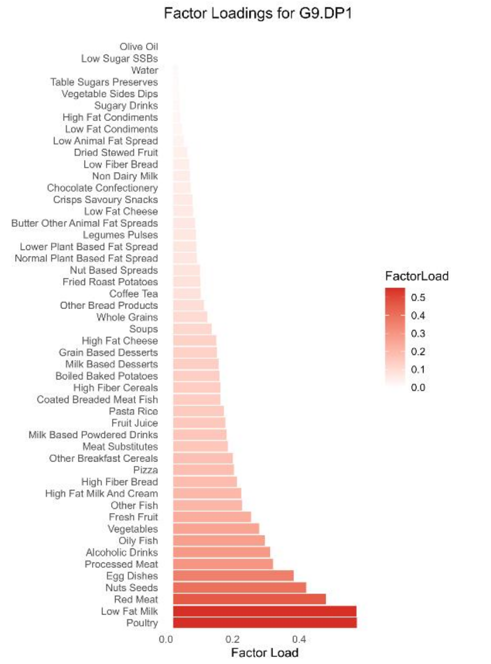

Supplementary Figure S6 | Factor Loadings for Food Groups in Dietary Patterns for Mineral-Rich, Moderate-Fat Diet

Supplementary Figure S7 | Factor Loadings for Food Groups in Dietary Patterns for Fiber-Enriched, Lipid-Conservative Diet

Supplementary Table S15

Food Groups	Case	P value	P for trend	HR (95% CI)
G1 DP1				
Q1	198	NA		Reference
Q2	185	0.535		0.938 [0.767, 1.147]
Q3	218	0.280		1.113 [0.916, 1.352]
Q4	249	**0.019**	0.007	**1.267 [1.039, 1.546]**
G1 DP2				
Q1	199	NA		Reference
Q2	197	0.720		1.037 [0.850, 1.264]
Q3	212	0.299		1.109 [0.913, 1.348]
Q4	242	**0.044**	0.033	**1.215 [1.005, 1.470]**
G2 DP1				
Q1	215	NA		Reference
Q2	186	0.362		0.907 [0.736, 1.118]
Q3	202	0.936		0.991 [0.791, 1.241]
Q4	247	0.186	0.137	1.194 [0.918, 1.552]
G2 DP2				
Q1	199	NA		Reference
Q2	187	0.713		0.963 [0.788, 1.177]
Q3	220	0.160		1.149 [0.947, 1.394]
Q4	244	**0.022**	0.006	**1.250 [1.033, 1.512]**
G3 DP1				
Q1	216	NA		Reference
Q2	193	0.186		0.868 [0.704, 1.070]
Q3	228	0.849		0.978 [0.782, 1.224]
Q4	213	0.221	0.438	0.844 [0.643, 1.108]
G3 DP2				
Q1	202	NA		Reference
Q2	184	0.442		0.924 [0.757, 1.130]
Q3	209	0.504		1.069 [0.879, 1.300]
Q4	255	**0.002**	0.001	**1.365 [1.121, 1.663]**
G4 DP1				
Q1	252	NA		Reference
Q2	200	**0.033**		**0.814 [0.673, 0.983]**
Q3	185	**0.003**		**0.742 [0.609, 0.904]**
Q4	213	**0.040**	0.026	**0.811 [0.663, 0.991]**
G4 DP2				

Q1	197	NA		Reference
Q2	204	0.359		1.098 [0.899, 1.342]
Q3	221	0.081		1.201 [0.977, 1.477]
Q4	228	**0.049**	0.036	**1.261 [1.001, 1.588]**
G5 DP1				
Q1	229	NA		Reference
Q2	195	0.190		0.877 [0.720, 1.067]
Q3	189	0.113		0.846 [0.688, 1.040]
Q4	237	0.978	0.971	1.003 [0.807, 1.246]
G5 DP2				
Q1	208	NA		Reference
Q2	167	0.044		0.810 [0.660, 0.994]
Q3	215	0.609		1.052 [0.866, 1.279]
Q4	260	**0.013**	0.002	**1.289 [1.055, 1.576]**
G6 DP1				
Q1	200	NA		Reference
Q2	215	0.326		0.908 [0.750, 1.100]
Q3	181	**0.003**		**0.730 [0.593, 0.899]**
Q4	224	0.070	0.023	0.820 [0.662, 1.016]
G6 DP2				
Q1	200	NA		Reference
Q2	187	0.730		0.965 [0.786, 1.184]
Q3	220	0.379		1.099 [0.890, 1.358]
Q4	243	0.173	0.111	1.186 [0.928, 1.515]
G7 DP1				
Q1	208	NA		Reference
Q2	195	0.423		0.917 [0.741, 1.134]
Q3	230	0.713		1.044 [0.829, 1.316]
Q4	217	0.423	0.710	0.890 [0.669, 1.184]
G7 DP2				
Q1	198	NA		Reference
Q2	179	0.390		0.915 [0.747, 1.121]
Q3	213	0.309		1.107 [0.910, 1.347]
Q4	260	**0.001**	<0.001	**1.377 [1.130, 1.677]**
G8 DP1				
Q1	211	NA		Reference
Q2	197	0.295		0.893 [0.722, 1.104]
Q3	217	0.655		0.948 [0.750, 1.198]

Q4	225	0.294	0.436	0.857 [0.642, 1.143]
G8 DP2				
Q1	203	NA		Reference
Q2	168	0.098		0.841 [0.685, 1.032]
Q3	221	0.220		1.128 [0.930, 1.368]
Q4	258	**0.003**	<0.001	**1.340 [1.105, 1.625]**
G9 DP1				
Q1	213	NA		Reference
Q2	210	0.472		0.928 [0.758, 1.137]
Q3	207	0.153		0.851 [0.683, 1.061]
Q4	220	0.085	0.068	0.797 [0.616, 1.032]

Supplementary Table S15 | Quartile Stratification of Dietary Patterns within Nutrient Groups Based on Z-Scores with Regression Outcomes Referenced to Quartile 1. G = Group, DP = Dietary Pattern.

All models adjusted for demographic and socio-economic covariates, including gender, age at recruitment, socio-economic status (Townsend deprivation index), employment status, educational attainment, health status, and log-transformed total caloric intake, lifestyle and health conditions, including average hours of sleep per night, current smoking status, Body Mass Index (BMI), current alcohol consumption status, physical activity level (IPAQ), Polygenic Risk Score (PRS), heart disease, hypertension, and diabetes.

Supplementary Table S16

	Gender			
	Male		Female	
	P	HR (95% CL)	P	HR (95% CL)
N total		66540		53169
N case		523		327
**Lipid-Rich, Calorically Dense Diet**				
G1 DP1	**0.023**	**1.082 [1.011, 1.158]**	0.117	1.072 [0.983, 1.169]
G2 DP1	**0.021**	**1.094 [1.014, 1.180]**	0.293	1.061 [0.950, 1.184]
G2 DP2	**0.011**	**1.128 [1.028, 1.238]**	0.052	1.127 [0.999, 1.272]
G3 DP2	**0.008**	**1.149 [1.037, 1.273]**	0.067	1.129 [0.992, 1.286]
G7 DP2	**0.012**	**1.117 [1.024, 1.217]**	**0.032**	**1.132 [1.011, 1.269]**
G8 DP2	**0.012**	**1.118 [1.025, 1.219]**	0.052	1.117 [0.999, 1.249]
**Hyper Glycemic, Fiber-Deficient Diet**				
G1 DP2	**0.007**	**1.156 [1.041, 1.285]**	0.175	1.114 [0.953, 1.303]
G5 DP1	0.867	1.005 [0.944, 1.071]	0.856	0.992 [0.912, 1.080]
G5 DP2	**<0.001**	**1.170 [1.075, 1.274]**	0.104	1.100 [0.981, 1.233]
G6 DP2	**0.03**	**1.142 [1.013, 1.288]**	0.611	1.042 [0.890, 1.219]
**Micronutrient-Abundant, Low-Lipid Diet**				
G4 DP1	0.067	0.934 [0.868, 1.005]	0.39	0.962 [0.881, 1.051]
G4 DP2	0.374	1.046 [0.947, 1.156]	0.297	1.073 [0.940, 1.224]
G6 DP1	0.072	0.934 [0.867, 1.006]	0.252	0.948 [0.864, 1.039]
**Mineral-Rich, Moderate-Fat Diet**				
G3 DP1	0.323	0.955 [0.873, 1.046]	0.832	0.987 [0.877, 1.112]
G7 DP1	0.108	0.925 [0.840, 1.017]	0.739	0.980 [0.867, 1.106]
G8 DP1	0.143	0.934 [0.853, 1.023]	0.905	0.993 [0.879, 1.121]
**Fiber-Enriched, Lipid-Conservative Diet**				
G9 DP1	**0.023**	**0.899 [0.820, 0.985]**	0.233	0.929 [0.823, 1.049]

Supplementary Table S16 | Stratified Analysis of Dietary Patterns for Each Health Focus Response Variable Combination and Their Association with ARC Based on Gender. G = Group, DP = Dietary Pattern.

All models adjusted for demographic and socio-economic covariates, including gender, age at recruitment, socio-economic status (Townsend deprivation index), employment status, educational attainment, health status, and log-transformed total caloric intake, lifestyle and health conditions, including average hours of sleep per night, current smoking status, Body Mass Index (BMI), current alcohol consumption status, physical activity level (IPAQ), Polygenic Risk Score (PRS), heart disease, hypertension, and diabetes.

Supplementary Table S17

	Age					
	< 50 Years		50-60 Years		> 60 Years	
	P	HR (95% CL)	P	HR (95% CL)	P	HR (95% CL)
N total		31680		45364		41815
N case		88		244		518
**Lipid-Rich, Calorically Dense Diet**						
G1 DP1	0.22	1.108 [0.941, 1.304]	**0.025**	**1.122 [1.014, 1.241]**	0.097	1.059 [0.990, 1.134]
G2 DP1	0.371	1.085 [0.907, 1.299]	0.23	1.073 [0.956, 1.205]	**0.036**	**1.090 [1.006, 1.182]**
G2 DP2	0.142	1.183 [0.945, 1.481]	**0.067**	**1.139 [0.991, 1.309]**	**0.018**	**1.121 [1.020, 1.232]**
G3 DP2	0.329	1.125 [0.888, 1.426]	**0.027**	**1.186 [1.020, 1.380]**	**0.018**	**1.133 [1.021, 1.257]**
G7 DP2	0.27	1.122 [0.915, 1.376]	**0.015**	**1.171 [1.031, 1.330]**	**0.026**	**1.106 [1.012, 1.208]**
G8 DP2	0.248	1.129 [0.919, 1.387]	**0.007**	**1.193 [1.049, 1.357]**	0.063	1.087 [0.995, 1.187]
**Hyper Glycemic, Fiber-Deficient Diet**						
G1 DP2	0.727	1.045 [0.817, 1.336]	0.097	1.148 [0.975, 1.352]	**0.011**	**1.160 [1.035, 1.300]**
G5 DP1	0.701	0.971 [0.834, 1.129]	0.428	0.962 [0.873, 1.059]	0.542	1.020 [0.956, 1.089]
G5 DP2	0.297	1.110 [0.913, 1.350]	**0.001**	**1.238 [1.091, 1.405]**	**0.017**	**1.114 [1.020, 1.217]**
G6 DP2	0.26	1.177 [0.887, 1.562]	0.155	1.137 [0.953, 1.356]	0.193	1.085 [0.960, 1.226]
**Micronutrient-Abundant, Low-Lipid Diet**						
G4 DP1	0.485	0.942 [0.796, 1.114]	**0.025**	**0.884 [0.793, 0.985]**	0.412	0.971 [0.904, 1.042]
G4 DP2	0.381	1.110 [0.879, 1.402]	0.212	1.098 [0.948, 1.273]	0.599	1.028 [0.928, 1.139]
G6 DP1	0.653	0.962 [0.813, 1.139]	**0.004**	**0.846 [0.755, 0.947]**	0.546	0.978 [0.908, 1.052]
**Mineral-Rich, Moderate-Fat Diet**						
G3 DP1	0.662	0.953 [0.769, 1.181]	**0.014**	**0.839 [0.730, 0.965]**	0.497	1.032 [0.943, 1.129]
G7 DP1	0.508	0.927 [0.742, 1.160]	**0.001**	**0.782 [0.676, 0.904]**	0.487	1.034 [0.941, 1.136]
G8 DP1	0.737	1.034 [0.852, 1.255]	**0.026**	**0.854 [0.744, 0.981]**	0.848	0.991 [0.902, 1.088]
**Fiber-Enriched, Lipid-Conservative Diet**						
G9 DP1	0.976	0.997 [0.821, 1.211]	**0.022**	**0.852 [0.742, 0.977]**	0.102	0.923 [0.839, 1.016]

Supplementary Table S17 | Stratified Analysis of Dietary Patterns for Each Health Focus Response Variable Combination and Their Association with ARC Based on Age. G = Group, DP = Dietary Pattern.

All models adjusted for demographic and socio-economic covariates, including gender, age at recruitment, socio-economic status (Townsend deprivation index), employment status, educational attainment, health status, and log-transformed total caloric intake, lifestyle and health conditions, including average hours of sleep per night, current smoking status, Body Mass Index (BMI), current alcohol consumption status, physical activity level (IPAQ), Polygenic Risk Score (PRS), heart disease, hypertension, and diabetes.

Supplementary Table S18

	Townsend deprivation index							
	Q1		Q2		Q3		Q4	
	P	HR (95% CL)	P	HR (95% CL)	P	HR (95% CL)	P	HR (95% CL)
N total		29853		29784		29630		29592
N case		192		203		199		256
**Lipid-Rich, Calorically Dense Diet**								
G1 DP1	0.238	1.073 [0.955, 1.205]	**0.01**	**1.163 [1.037, 1.305]**	0.898	1.007 [0.905, 1.121]	**0.071**	**1.089 [0.993, 1.195]**
G2 DP1	0.308	1.072 [0.938, 1.224]	0.364	1.063 [0.931, 1.214]	0.943	1.005 [0.882, 1.145]	**0.005**	**1.163 [1.046, 1.294]**
G2 DP2	0.493	1.058 [0.901, 1.241]	**0.001**	**1.305 [1.113, 1.530]**	0.515	1.050 [0.907, 1.216]	**0.053**	**1.136 [0.998, 1.293]**
G3 DP2	0.233	1.113 [0.933, 1.326]	**0.001**	**1.329 [1.119, 1.579]**	0.412	1.070 [0.911, 1.257]	0.145	1.109 [0.965, 1.275]
G7 DP2	0.271	1.087 [0.937, 1.261]	**0.002**	**1.256 [1.087, 1.452]**	0.366	1.066 [0.928, 1.225]	**0.09**	**1.109 [0.984, 1.251]**
G8 DP2	0.295	1.082 [0.933, 1.255]	**0.002**	**1.253 [1.084, 1.449]**	0.334	1.071 [0.932, 1.229]	0.133	1.096 [0.973, 1.236]
**Hyper Glycemic, Fiber-Deficient Diet**								
G1 DP2	0.843	0.980 [0.803, 1.196]	**0.036**	**1.214 [1.012, 1.456]**	**0.052**	**1.192 [0.998, 1.424]**	**0.045**	**1.166 [1.003, 1.354]**
G5 DP1	0.467	0.959 [0.856, 1.074]	0.204	0.931 [0.835, 1.039]	0.112	1.084 [0.981, 1.197]	0.845	1.009 [0.924, 1.101]
G5 DP2	0.263	1.091 [0.937, 1.270]	**0.003**	**1.241 [1.078, 1.429]**	**0.042**	**1.156 [1.005, 1.329]**	0.059	1.119 [0.996, 1.257]
G6 DP2	0.492	1.074 [0.876, 1.316]	0.135	1.165 [0.954, 1.423]	0.494	1.070 [0.881, 1.301]	0.166	1.124 [0.952, 1.327]
**Micronutrient-Abundant, Low-Lipid Diet**								
G4 DP1	0.485	0.957 [0.846, 1.082]	**0.003**	**0.826 [0.729, 0.936]**	0.882	0.992 [0.887, 1.108]	0.639	0.977 [0.889, 1.075]
G4 DP2	0.771	1.026 [0.865, 1.216]	0.592	1.047 [0.886, 1.236]	0.43	1.070 [0.905, 1.264]	0.284	1.078 [0.939, 1.238]
G6 DP1	0.254	0.928 [0.817, 1.055]	**0.004**	**0.830 [0.730, 0.942]**	0.957	0.997 [0.889, 1.118]	0.633	0.976 [0.885, 1.077]
**Mineral-Rich, Moderate-Fat Diet**								
G3 DP1	0.834	0.984 [0.842, 1.149]	**0.048**	**0.854 [0.731, 0.999]**	0.921	1.007 [0.872, 1.163]	0.94	1.005 [0.888, 1.137]
G7 DP1	0.673	0.966 [0.821, 1.136]	**0.011**	**0.809 [0.687, 0.953]**	0.763	0.977 [0.839, 1.137]	0.943	1.005 [0.884, 1.141]
G8 DP1	0.437	0.939 [0.801, 1.101]	**0.019**	**0.828 [0.707, 0.969]**	0.588	0.960 [0.829, 1.112]	0.394	1.054 [0.934, 1.190]
**Fiber-Enriched, Lipid-Conservative Diet**								
G9 DP1	0.258	0.912 [0.777, 1.070]	**0.006**	**0.803 [0.686, 0.939]**	0.143	0.895 [0.771, 1.038]	0.94	1.005 [0.887, 1.138]

Supplementary Table S18 | Quartile-Based Stratified Analysis of Dietary Patterns for Each Health Focus Response Variable Combination and Their Association with ARC Based on Townsend deprivation index. G = Group, DP = Dietary Pattern.

All models adjusted for demographic and socio-economic covariates, including gender, age at recruitment, socio-economic status (Townsend deprivation index), employment status, educational attainment, health status, and log-transformed total caloric intake, lifestyle and health conditions, including average hours of sleep per night, current smoking status, Body Mass Index (BMI), current alcohol consumption status, physical activity level (IPAQ), Polygenic Risk Score (PRS), heart disease, hypertension, and diabetes.

Supplementary Table S19

	Education score							
	Q1		Q2		Q3		Q4	
	P	HR (95% CL)	P	HR (95% CL)	P	HR (95% CL)	P	HR (95% CL)
N total		29796		29719		29666		29678
N case		152		226		224		248
**Lipid-Rich, Calorically Dense Diet**								
G1 DP1	0.099	1.117 [0.979, 1.274]	0.351	1.051 [0.946, 1.168]	**0.021**	**1.133 [1.019, 1.260]**	0.332	1.047 [0.954, 1.149]
G2 DP1	0.125	1.121 [0.969, 1.298]	0.432	1.051 [0.928, 1.191]	0.286	1.069 [0.945, 1.209]	0.097	1.099 [0.983, 1.228]
G2 DP2	0.053	1.193 [0.998, 1.426]	0.394	1.065 [0.922, 1.230]	**0.003**	**1.249 [1.077, 1.447]**	0.325	1.067 [0.937, 1.215]
G3 DP2	0.06	1.211 [0.992, 1.479]	0.356	1.078 [0.919, 1.265]	**0.004**	**1.271 [1.082, 1.493]**	0.284	1.079 [0.939, 1.239]
G7 DP2	0.069	1.173 [0.988, 1.392]	0.245	1.084 [0.946, 1.243]	**0.008**	**1.200 [1.049, 1.373]**	0.192	1.083 [0.961, 1.219]
G8 DP2	0.078	1.167 [0.983, 1.386]	0.313	1.072 [0.936, 1.228]	**0.009**	**1.196 [1.045, 1.369]**	0.184	1.084 [0.963, 1.221]
**Hyper Glycemic, Fiber-Deficient Diet**								
G1 DP2	0.27	1.131 [0.909, 1.407]	0.16	1.134 [0.951, 1.353]	0.224	1.113 [0.937, 1.322]	**0.039**	**1.173 [1.008, 1.363]**
G5 DP1	0.813	0.985 [0.869, 1.117]	0.884	0.993 [0.898, 1.097]	0.546	0.969 [0.876, 1.072]	0.467	1.033 [0.946, 1.129]
G5 DP2	0.146	1.134 [0.957, 1.344]	0.22	1.092 [0.949, 1.256]	**0.001**	**1.245 [1.090, 1.421]**	0.051	1.122 [0.999, 1.261]
G6 DP2	0.805	1.030 [0.817, 1.297]	0.406	1.083 [0.897, 1.307]	**0.016**	**1.261 [1.044, 1.524]**	0.494	1.060 [0.897, 1.252]
**Micronutrient-Abundant, Low-Lipid Diet**								
G4 DP1	0.296	0.928 [0.806, 1.068]	0.536	0.966 [0.865, 1.078]	**0.031**	**0.882 [0.788, 0.988]**	0.693	0.981 [0.891, 1.080]
G4 DP2	0.43	1.080 [0.892, 1.308]	0.921	1.008 [0.861, 1.180]	0.709	1.030 [0.881, 1.204]	0.144	1.111 [0.965, 1.278]
G6 DP1	0.124	0.892 [0.771, 1.032]	0.661	0.975 [0.870, 1.092]	0.121	0.913 [0.815, 1.024]	0.334	0.951 [0.860, 1.053]
**Mineral-Rich, Moderate-Fat Diet**								
G3 DP1	0.491	0.942 [0.795, 1.116]	0.602	1.038 [0.902, 1.195]	0.383	0.939 [0.816, 1.081]	0.377	0.943 [0.827, 1.075]
G7 DP1	0.426	0.929 [0.775, 1.114]	0.565	0.957 [0.825, 1.111]	0.215	0.911 [0.787, 1.055]	0.649	0.969 [0.848, 1.108]
G8 DP1	0.398	0.927 [0.777, 1.106]	0.381	0.937 [0.809, 1.084]	0.287	0.926 [0.803, 1.067]	0.935	1.005 [0.886, 1.140]
**Fiber-Enriched, Lipid-Conservative Diet**								
G9 DP1	0.096	0.859 [0.719, 1.027]	0.317	0.929 [0.803, 1.074]	0.087	0.882 [0.765, 1.018]	0.402	0.946 [0.832, 1.076]

Supplementary Table S19 | Quartile-Based Stratified Analysis of Dietary Patterns for Each Health Focus Response Variable Combination and Their Association with ARC Based on Education score. G = Group, DP = Dietary Pattern.

All models adjusted for demographic and socio-economic covariates, including gender, age at recruitment, socio-economic status (Townsend deprivation index), employment status, educational attainment, health status, and log-transformed total caloric intake, lifestyle and health conditions, including average hours of sleep per night, current smoking status, Body Mass Index (BMI), current alcohol consumption status, physical activity level (IPAQ), Polygenic Risk Score (PRS), heart disease, hypertension, and diabetes.

Supplementary Table S20

	BMI							
	Q1		Q2		Q3		Q4	
	P	HR (95% CL)	P	HR (95% CL)	P	HR (95% CL)	P	HR (95% CL)
N total	29801		29753		29703		29602	
N case	140		162		223		325	
**Lipid-Rich, Calorically Dense Diet**								
G1 DP1	**0.007**	**1.207 [1.053, 1.385]**	0.772	1.019 [0.900, 1.153]	0.318	1.055 [0.950, 1.173]	0.066	1.081 [0.995, 1.175]
G2 DP1	**0.004**	**1.258 [1.077, 1.470]**	0.595	1.042 [0.896, 1.211]	0.932	0.994 [0.875, 1.130]	0.055	1.098 [0.998, 1.207]
G2 DP2	**0.022**	**1.244 [1.032, 1.499]**	0.464	0.941 [0.801, 1.107]	**0.013**	**1.211 [1.042, 1.408]**	**0.021**	**1.146 [1.020, 1.288]**
G3 DP2	**0.006**	**1.346 [1.088, 1.665]**	0.466	0.935 [0.779, 1.121]	**0.039**	**1.185 [1.009, 1.393]**	**0.02**	**1.160 [1.024, 1.314]**
G7 DP2	**0.002**	**1.338 [1.115, 1.607]**	0.713	0.971 [0.829, 1.137]	0.112	1.117 [0.975, 1.280]	**0.019**	**1.134 [1.021, 1.260]**
G8 DP2	**0.003**	**1.325 [1.103, 1.590]**	0.72	0.972 [0.830, 1.137]	0.137	1.108 [0.968, 1.270]	**0.023**	**1.130 [1.017, 1.256]**
**Hyper Glycemic, Fiber-Deficient Diet**								
G1 DP2	0.115	1.190 [0.959, 1.479]	0.847	0.980 [0.794, 1.209]	0.14	1.140 [0.958, 1.356]	**0.01**	**1.196 [1.045, 1.370]**
G5 DP1	0.369	0.942 [0.826, 1.074]	0.57	0.965 [0.854, 1.091]	0.651	1.023 [0.926, 1.131]	0.684	1.016 [0.940, 1.099]
G5 DP2	**0.002**	**1.288 [1.095, 1.514]**	0.809	1.020 [0.871, 1.194]	0.07	1.133 [0.990, 1.296]	**0.009**	**1.156 [1.037, 1.288]**
G6 DP2	**0.014**	**1.363 [1.064, 1.747]**	0.465	0.922 [0.740, 1.147]	0.414	1.082 [0.896, 1.306]	0.081	1.140 [0.984, 1.322]
**Micronutrient-Abundant, Low-Lipid Diet**								
G4 DP1	**0.025**	**0.844 [0.728, 0.979]**	0.37	1.058 [0.935, 1.197]	0.308	0.943 [0.842, 1.056]	0.102	0.929 [0.850, 1.015]
G4 DP2	0.065	1.211 [0.988, 1.485]	0.914	0.990 [0.820, 1.194]	0.467	1.060 [0.906, 1.241]	0.596	1.034 [0.914, 1.169]
G6 DP1	**0.016**	**0.830 [0.714, 0.966]**	0.739	1.023 [0.897, 1.166]	0.339	0.945 [0.842, 1.061]	0.19	0.941 [0.860, 1.031]
**Mineral-Rich, Moderate-Fat Diet**								
G3 DP1	0.262	0.902 [0.754, 1.080]	0.424	0.934 [0.791, 1.104]	0.582	0.961 [0.833, 1.108]	0.761	1.018 [0.909, 1.139]
G7 DP1	0.131	0.864 [0.715, 1.045]	0.577	0.951 [0.799, 1.133]	0.632	0.964 [0.831, 1.119]	0.585	0.968 [0.860, 1.089]
G8 DP1	0.595	0.950 [0.785, 1.149]	0.652	0.961 [0.808, 1.143]	0.536	0.956 [0.827, 1.104]	0.441	0.957 [0.857, 1.070]
**Fiber-Enriched, Lipid-Conservative Diet**								
G9 DP1	0.182	0.879 [0.728, 1.062]	0.827	0.981 [0.829, 1.162]	0.094	0.883 [0.763, 1.021]	0.119	0.914 [0.816, 1.023]

Supplementary Table S20 | Quartile-Based Stratified Analysis of Dietary Patterns for Each Health Focus Response Variable Combination and Their Association with ARC Based on BMI. G = Group, DP = Dietary Pattern.

All models adjusted for demographic and socio-economic covariates, including gender, age at recruitment, socio-economic status (Townsend deprivation index), employment status, educational attainment, health status, and log-transformed total caloric intake, lifestyle and health conditions, including average hours of sleep per night, current smoking status, Body Mass Index (BMI), current alcohol consumption status, physical activity level (IPAQ), Polygenic Risk Score (PRS), heart disease, hypertension, and diabetes.

Supplementary Table S21

	Smoke status					
	Previous		Current		Never	
	P	HR (95% CL)	P	HR (95% CL)	P	HR (95% CL)
N total	42886		68472		8351	
N case	386		388		76	
**Lipid-Rich, Calorically Dense Diet**						
G1 DP1	**0.005**	**1.120 [1.035, 1.213]**	0.803	1.010 [0.934, 1.092]	**0.048**	**1.202 [1.001, 1.443]**
G2 DP1	0.057	1.092 [0.998, 1.195]	0.509	1.033 [0.938, 1.138]	**0.029**	**1.231 [1.021, 1.485]**
G2 DP2	**0.002**	**1.197 [1.071, 1.337]**	0.467	1.041 [0.935, 1.158]	0.122	1.217 [0.949, 1.562]
G3 DP2	**0.001**	**1.226 [1.086, 1.383]**	0.623	1.030 [0.916, 1.158]	0.065	1.291 [0.984, 1.694]
G7 DP2	**0.003**	**1.168 [1.055, 1.292]**	0.339	1.051 [0.949, 1.163]	0.085	1.221 [0.973, 1.532]
G8 DP2	**0.003**	**1.168 [1.055, 1.294]**	0.386	1.046 [0.945, 1.157]	0.124	1.196 [0.952, 1.504]
**Hyper Glycemic, Fiber-Deficient Diet**						
G1 DP2	0.182	1.094 [0.959, 1.247]	**0.021**	**1.169 [1.024, 1.334]**	0.176	1.191 [0.924, 1.535]
G5 DP1	0.265	0.957 [0.886, 1.034]	0.182	1.052 [0.977, 1.132]	0.803	0.979 [0.829, 1.156]
G5 DP2	**0.001**	**1.178 [1.065, 1.303]**	0.137	1.083 [0.975, 1.202]	0.07	1.206 [0.985, 1.476]
G6 DP2	**0.023**	**1.177 [1.023, 1.353]**	0.765	1.022 [0.887, 1.176]	0.387	1.149 [0.839, 1.572]
**Micronutrient-Abundant, Low-Lipid Diet**						
G4 DP1	**0.003**	**0.875 [0.803, 0.954]**	0.924	1.004 [0.926, 1.088]	0.845	1.018 [0.854, 1.214]
G4 DP2	0.385	1.053 [0.938, 1.181]	0.775	1.018 [0.902, 1.148]	0.092	1.247 [0.965, 1.612]
G6 DP1	**0.004**	**0.876 [0.802, 0.958]**	0.859	1.008 [0.927, 1.095]	0.574	0.947 [0.784, 1.144]
**Mineral-Rich, Moderate-Fat Diet**						
G3 DP1	0.213	0.934 [0.838, 1.040]	0.928	0.995 [0.895, 1.106]	0.768	1.036 [0.820, 1.308]
G7 DP1	0.087	0.906 [0.810, 1.014]	0.689	0.978 [0.875, 1.092]	0.799	1.032 [0.810, 1.316]
G8 DP1	**0.035**	**0.890 [0.798, 0.992]**	0.926	0.995 [0.894, 1.108]	0.318	1.121 [0.896, 1.403]
**Fiber-Enriched, Lipid-Conservative Diet**						
G9 DP1	**0.007**	**0.860 [0.771, 0.960]**	0.499	0.964 [0.866, 1.073]	0.654	0.947 [0.746, 1.202]

Supplementary Table S21 | Stratified Analysis of Dietary Patterns for Each Health Focus Response Variable Combination and Their Association with ARC Based on Smoking status. G = Group, DP = Dietary Pattern.

All models adjusted for demographic and socio-economic covariates, including gender, age at recruitment, socio-economic status (Townsend deprivation index), employment status, educational attainment, health status, and log-transformed total caloric intake, lifestyle and health conditions, including average hours of sleep per night, current smoking status, Body Mass Index (BMI), current alcohol consumption status, physical activity level (IPAQ), Polygenic Risk Score (PRS), heart disease, hypertension, and diabetes.

Supplementary Table S22

	Alcohol status					
	Previous		Current		Never	
	P	HR (95% CL)	P	HR (95% CL)	P	HR (95% CL)
N total	3466		112832		3411	
N case	46		768		36	
**Lipid-Rich, Calorically Dense Diet**						
G1 DP1	0.242	1.122 [0.925, 1.360]	**0.013**	**1.074 [1.015, 1.137]**	0.189	1.179 [0.922, 1.507]
G2 DP1	0.259	1.144 [0.905, 1.446]	0.066	1.064 [0.996, 1.137]	0.095	1.288 [0.956, 1.735]
G2 DP2	0.158	1.226 [0.924, 1.626]	**0.004**	**1.122 [1.038, 1.213]**	0.488	1.121 [0.812, 1.546]
G3 DP2	0.092	1.298 [0.958, 1.758]	**0.006**	**1.127 [1.035, 1.228]**	0.247	1.233 [0.865, 1.757]
G7 DP2	0.256	1.158 [0.899, 1.490]	**0.003**	**1.115 [1.037, 1.199]**	0.161	1.249 [0.915, 1.705]
G8 DP2	0.218	1.173 [0.910, 1.512]	**0.004**	**1.111 [1.034, 1.195]**	0.198	1.223 [0.900, 1.661]
**Hyper Glycemic, Fiber-Deficient Diet**						
G1 DP2	0.148	1.247 [0.925, 1.682]	**0.041**	**1.104 [1.004, 1.214]**	0.164	1.300 [0.899, 1.880]
G5 DP1	0.684	1.036 [0.874, 1.228]	0.71	0.990 [0.937, 1.045]	0.441	0.909 [0.714, 1.158]
G5 DP2	**0.031**	**1.296 [1.024, 1.641]**	**0.002**	**1.124 [1.044, 1.209]**	0.221	1.187 [0.902, 1.563]
G6 DP2	0.354	1.175 [0.835, 1.654]	0.103	1.087 [0.983, 1.202]	0.072	1.537 [0.962, 2.454]
**Micronutrient-Abundant, Low-Lipid Diet**						
G4 DP1	0.541	0.938 [0.766, 1.150]	0.067	0.946 [0.891, 1.004]	0.143	0.816 [0.621, 1.072]
G4 DP2	0.283	1.179 [0.873, 1.591]	0.342	1.042 [0.958, 1.133]	0.305	1.223 [0.832, 1.798]
G6 DP1	0.612	0.947 [0.768, 1.168]	**0.044**	**0.938 [0.882, 0.998]**	0.234	0.852 [0.653, 1.110]
**Mineral-Rich, Moderate-Fat Diet**						
G3 DP1	0.607	0.929 [0.704, 1.228]	0.24	0.955 [0.885, 1.031]	0.673	1.067 [0.791, 1.438]
G7 DP1	0.667	0.941 [0.712, 1.243]	0.11	0.937 [0.865, 1.015]	0.821	0.965 [0.707, 1.316]
G8 DP1	0.977	0.996 [0.765, 1.298]	0.148	0.945 [0.875, 1.020]	0.803	0.961 [0.705, 1.311]
**Fiber-Enriched, Lipid-Conservative Diet**						
G9 DP1	0.962	0.994 [0.762, 1.295]	**0.004**	**0.893 [0.826, 0.965]**	0.532	1.103 [0.811, 1.499]

Supplementary Table S22 | Stratified Analysis of Dietary Patterns for Each Health Focus Response Variable Combination and Their Association with ARC Based on Alcohol status. G = Group, DP = Dietary Pattern.

All models adjusted for demographic and socio-economic covariates, including gender, age at recruitment, socio-economic status (Townsend deprivation index), employment status, educational attainment, health status, and log-transformed total caloric intake, lifestyle and health conditions, including average hours of sleep per night, current smoking status, Body Mass Index (BMI), current alcohol consumption status, physical activity level (IPAQ), Polygenic Risk Score (PRS), heart disease, hypertension, and diabetes.

Supplementary Table S23

	Physical activity (IPAQ)					
	High		Moderate		Low	
	P	HR (95% CL)	P	HR (95% CL)	P	HR (95% CL)
N total		50453		47128		22128
N case		357		299		194
**Lipid-Rich, Calorically Dense Diet**						
G1 DP1	**0.017**	**1.101 [1.018, 1.192]**	0.242	1.057 [0.963, 1.159]	0.287	1.063 [0.950, 1.190]
G2 DP1	**0.011**	**1.127 [1.028, 1.235]**	0.492	1.039 [0.931, 1.160]	0.412	1.056 [0.928, 1.201]
G2 DP2	0.116	1.091 [0.979, 1.216]	0.175	1.092 [0.962, 1.240]	**0.003**	**1.271 [1.083, 1.492]**
G3 DP2	**0.043**	**1.130 [1.004, 1.273]**	0.227	1.090 [0.948, 1.253]	**0.012**	**1.247 [1.050, 1.480]**
G7 DP2	**0.017**	**1.132 [1.022, 1.253]**	0.191	1.083 [0.961, 1.221]	**0.045**	**1.158 [1.003, 1.337]**
G8 DP2	**0.035**	**1.115 [1.008, 1.234]**	0.173	1.087 [0.964, 1.225]	**0.037**	**1.167 [1.010, 1.349]**
**Hyper Glycemic, Fiber-Deficient Diet**						
G1 DP2	0.611	1.036 [0.905, 1.186]	0.302	1.085 [0.930, 1.266]	**<0.001**	**1.404 [1.189, 1.659]**
G5 DP1	0.24	0.955 [0.883, 1.031]	0.945	0.997 [0.912, 1.089]	0.095	1.090 [0.985, 1.207]
G5 DP2	**0.029**	**1.121 [1.012, 1.241]**	0.101	1.105 [0.981, 1.246]	**0.002**	**1.245 [1.084, 1.429]**
G6 DP2	**0.047**	**1.154 [1.002, 1.330]**	0.561	1.050 [0.891, 1.237]	0.343	1.102 [0.901, 1.347]
**Micronutrient-Abundant, Low-Lipid Diet**						
G4 DP1	0.272	0.955 [0.879, 1.037]	0.388	0.958 [0.870, 1.056]	0.098	0.900 [0.794, 1.020]
G4 DP2	0.099	1.105 [0.981, 1.244]	0.986	1.001 [0.872, 1.149]	0.599	1.046 [0.885, 1.235]
G6 DP1	0.179	0.943 [0.865, 1.027]	0.373	0.955 [0.864, 1.056]	0.144	0.911 [0.803, 1.033]
**Mineral-Rich, Moderate-Fat Diet**						
G3 DP1	0.843	0.989 [0.888, 1.102]	0.533	0.961 [0.849, 1.088]	0.438	0.942 [0.809, 1.096]
G7 DP1	0.629	0.973 [0.869, 1.089]	0.343	0.939 [0.825, 1.069]	0.261	0.913 [0.779, 1.070]
G8 DP1	0.687	1.022 [0.918, 1.139]	0.281	0.933 [0.823, 1.058]	0.084	0.874 [0.750, 1.018]
**Fiber-Enriched, Lipid-Conservative Diet**						
G9 DP1	0.37	0.951 [0.852, 1.061]	0.135	0.908 [0.800, 1.030]	**0.041**	**0.852 [0.730, 0.993]**

Supplementary Table S23 | Stratified Analysis of Dietary Patterns for Each Health Focus Response Variable Combination and Their Association with ARC Based on Physical activity level. G = Group, DP = Dietary Pattern.

All models adjusted for demographic and socio-economic covariates, including gender, age at recruitment, socio-economic status (Townsend deprivation index), employment status, educational attainment, health status, and log-transformed total caloric intake, lifestyle and health conditions, including average hours of sleep per night, current smoking status, Body Mass Index (BMI), current alcohol consumption status, physical activity level (IPAQ), Polygenic Risk Score (PRS), heart disease, hypertension, and diabetes.

Supplementary Table S24

	Diabetes			
	No		Yes	
	P	HR (95% CL)	P	HR (95% CL)
N total		111994		7715
N case		686		164
**Lipid-Rich, Calorically Dense Diet**				
G1 DP1	**0.002**	**1.101 [1.037, 1.169]**	0.992	1.001 [0.892, 1.123]
G2 DP1	**0.021**	**1.085 [1.012, 1.164]**	0.297	1.076 [0.937, 1.236]
G2 DP2	**0.001**	**1.158 [1.066, 1.258]**	0.699	1.032 [0.879, 1.212]
G3 DP2	**0.001**	**1.168 [1.067, 1.279]**	0.491	1.063 [0.893, 1.265]
G7 DP2	**0.001**	**1.145 [1.060, 1.236]**	0.504	1.052 [0.907, 1.220]
G8 DP2	**0.001**	**1.142 [1.057, 1.233]**	0.6	1.040 [0.898, 1.205]
**Hyper Glycemic, Fiber-Deficient Diet**				
G1 DP2	**0.049**	**1.104 [1.000, 1.219]**	**0.007**	**1.289 [1.070, 1.553]**
G5 DP1	0.346	0.973 [0.918, 1.030]	0.071	1.101 [0.992, 1.223]
G5 DP2	**<0.001**	**1.149 [1.065, 1.239]**	0.092	1.140 [0.979, 1.327]
G6 DP2	**0.021**	**1.133 [1.019, 1.260]**	0.915	1.011 [0.821, 1.246]
**Micronutrient-Abundant, Low-Lipid Diet**				
G4 DP1	**0.01**	**0.920 [0.863, 0.980]**	0.515	1.040 [0.924, 1.171]
G4 DP2	0.207	1.059 [0.969, 1.157]	0.56	1.054 [0.884, 1.256]
G6 DP1	**0.013**	**0.921 [0.862, 0.983]**	0.844	1.013 [0.894, 1.147]
**Mineral-Rich, Moderate-Fat Diet**				
G3 DP1	0.175	0.946 [0.873, 1.025]	0.488	1.057 [0.904, 1.235]
G7 DP1	0.081	0.928 [0.853, 1.009]	0.786	1.023 [0.869, 1.203]
G8 DP1	0.142	0.941 [0.867, 1.021]	0.821	1.018 [0.872, 1.188]
**Fiber-Enriched, Lipid-Conservative Diet**				
G9 DP1	**0.009**	**0.896 [0.825, 0.973]**	0.725	0.972 [0.829, 1.139]

Supplementary Table S24 | Stratified Analysis of Dietary Patterns for Each Health Focus Response Variable Combination and Their Association with ARC Based on diabetes. G = Group, DP = Dietary Pattern.

All models adjusted for demographic and socio-economic covariates, including gender, age at recruitment, socio-economic status (Townsend deprivation index), employment status, educational attainment, health status, and log-transformed total caloric intake, lifestyle and health conditions, including average hours of sleep per night, current smoking status, Body Mass Index (BMI), current alcohol consumption status, physical activity level (IPAQ), Polygenic Risk Score (PRS), heart disease, hypertension, and diabetes.

Supplementary Table S25

	Cardiovascular disease			
	No		Yes	
	P	HR (95% CL)	P	HR (95% CL)
N total		84956		34753
N case		288		562
**Lipid-Rich, Calorically Dense Diet**				
G1 DP1	**0.034**	**1.107 [1.008, 1.216]**	**0.044**	**1.068 [1.002, 1.140]**
G2 DP1	0.336	1.055 [0.946, 1.178]	**0.017**	**1.096 [1.017, 1.182]**
G2 DP2	0.071	1.127 [0.990, 1.282]	**0.006**	**1.134 [1.037, 1.240]**
G3 DP2	**0.045**	**1.155 [1.003, 1.331]**	**0.007**	**1.143 [1.037, 1.260]**
G7 DP2	**0.006**	**1.187 [1.051, 1.339]**	**0.026**	**1.099 [1.011, 1.194]**
G8 DP2	**0.006**	**1.188 [1.052, 1.341]**	**0.037**	**1.092 [1.005, 1.186]**
**Hyper Glycemic, Fiber-Deficient Diet**				
G1 DP2	0.391	1.071 [0.915, 1.253]	**0.003**	**1.176 [1.059, 1.306]**
G5 DP1	0.275	0.951 [0.868, 1.041]	0.488	1.022 [0.961, 1.086]
G5 DP2	**0.022**	**1.151 [1.021, 1.298]**	**0.001**	**1.149 [1.058, 1.248]**
G6 DP2	0.12	1.141 [0.966, 1.347]	0.124	1.095 [0.976, 1.228]
**Micronutrient-Abundant, Low-Lipid Diet**				
G4 DP1	0.055	0.906 [0.820, 1.002]	0.233	0.960 [0.897, 1.027]
G4 DP2	0.152	1.106 [0.963, 1.269]	0.466	1.037 [0.941, 1.142]
G6 DP1	**0.024**	**0.888 [0.801, 0.984]**	0.264	0.961 [0.896, 1.031]
**Mineral-Rich, Moderate-Fat Diet**				
G3 DP1	0.188	0.918 [0.808, 1.043]	0.813	0.990 [0.907, 1.079]
G7 DP1	0.06	0.880 [0.770, 1.006]	0.615	0.977 [0.893, 1.070]
G8 DP1	0.354	0.941 [0.829, 1.070]	0.381	0.961 [0.881, 1.050]
**Fiber-Enriched, Lipid-Conservative Diet**				
G9 DP1	0.052	0.881 [0.775, 1.001]	0.086	0.925 [0.846, 1.011]

Supplementary Table S25 | Stratified Analysis of Dietary Patterns for Each Health Focus Response Variable Combination and Their Association with ARC Based on Cardiovascular disease. G = Group, DP = Dietary Pattern.

All models adjusted for demographic and socio-economic covariates, including gender, age at recruitment, socio-economic status (Townsend deprivation index), employment status, educational attainment, health status, and log-transformed total caloric intake, lifestyle and health conditions, including average hours of sleep per night, current smoking status, Body Mass Index (BMI), current alcohol consumption status, physical activity level (IPAQ), Polygenic Risk Score (PRS), heart disease, hypertension, and diabetes.

Supplementary Table S26

	Hypertension			
	No		Yes	
	P	HR (95% CL)	P	HR (95% CL)
N total		87513		32196
N case		329		521
**Lipid-Rich, Calorically Dense Diet**				
G1 DP1	**0.035**	**1.097 [1.006, 1.196]**	0.053	1.069 [0.999, 1.144]
G2 DP1	**0.003**	**1.160 [1.052, 1.279]**	0.347	1.039 [0.959, 1.126]
G2 DP2	0.167	1.086 [0.966, 1.221]	**0.002**	**1.161 [1.056, 1.276]**
G3 DP2	0.064	1.130 [0.993, 1.287]	**0.006**	**1.153 [1.041, 1.277]**
G7 DP2	**0.022**	**1.140 [1.019, 1.276]**	**0.014**	**1.115 [1.022, 1.215]**
G8 DP2	**0.032**	**1.130 [1.010, 1.264]**	**0.015**	**1.113 [1.021, 1.213]**
**Hyper Glycemic, Fiber-Deficient Diet**				
G1 DP2	0.123	1.118 [0.970, 1.289]	**0.008**	**1.159 [1.039, 1.294]**
G5 DP1	0.944	1.003 [0.924, 1.089]	0.974	0.999 [0.937, 1.065]
G5 DP2	**0.016**	**1.145 [1.025, 1.278]**	**0.002**	**1.147 [1.052, 1.251]**
G6 DP2	**0.027**	**1.191 [1.020, 1.390]**	0.362	1.057 [0.938, 1.191]
**Micronutrient-Abundant, Low-Lipid Diet**				
G4 DP1	0.258	0.949 [0.867, 1.039]	0.098	0.941 [0.876, 1.011]
G4 DP2	0.159	1.096 [0.965, 1.245]	0.523	1.033 [0.934, 1.142]
G6 DP1	0.424	0.963 [0.878, 1.056]	**0.036**	**0.923 [0.857, 0.995]**
**Mineral-Rich, Moderate-Fat Diet**				
G3 DP1	0.918	0.994 [0.888, 1.113]	0.277	0.950 [0.866, 1.042]
G7 DP1	0.887	0.991 [0.881, 1.116]	0.083	0.918 [0.834, 1.011]
G8 DP1	0.426	1.047 [0.935, 1.173]	**0.029**	**0.901 [0.821, 0.989]**
**Fiber-Enriched, Lipid-Conservative Diet**				
G9 DP1	0.998	1.000 [0.892, 1.120]	**0.001**	**0.856 [0.779, 0.941]**

Supplementary Table S26 | Stratified Analysis of Dietary Patterns for Each Health Focus Response Variable Combination and Their Association with ARC Based on hypertension. G = Group, DP = Dietary Pattern.

All models adjusted for demographic and socio-economic covariates, including gender, age at recruitment, socio-economic status (Townsend deprivation index), employment status, educational attainment, health status, and log-transformed total caloric intake, lifestyle and health conditions, including average hours of sleep per night, current smoking status, Body Mass Index (BMI), current alcohol consumption status, physical activity level (IPAQ), Polygenic Risk Score (PRS), heart disease, hypertension, and diabetes.

Supplementary Table S27

	Latent Profile divided by food groups								
**Characteristics**	X1	X2	X3	X4	X5	X6	X7	X8	p
N	33857	15537	14636	12677	3342	29309	6308	3872	
Ncase	**209 (0.62)**	**121 (0.78)**	**97 (0.66)**	**61 (0.48)**	**26 (0.75)**	**249 (0.85)**	**52 (0.82)**	**23 (0.59)**	0.001
Male, n (%)	15646 (46.2)	8524 (54.9)	3869 (26.4)	4968 (39.2)	1240 (37.1)	14327 (48.9)	3180 (50.4)	1329 (34.3)	<0.001
TDI[a]	-1.79 (2.76)	-1.52 (2.90)	-1.73 (2.77)	-1.44 (2.94)	-1.43 (2.92)	-1.74 (2.79)	-1.37 (2.96)	-0.98 (3.05)	<0.001
Age (years) [a]	56.72 (7.70)	56.19 (7.84)	56.50 (7.59)	55.36 (7.90)	54.50 (7.99)	56.26 (7.86)	53.64 (8.03)	56.05 (7.78)	<0.001
Education score[a]	9.97 (12.36)	12.05 (14.12)	10.46 (12.77)	10.54 (12.54)	11.82 (13.42)	13.12 (14.68)	13.54 (15.07)	10.40 (12.50)	<0.001
Employment score[a]	0.75 (3.54)	0.85 (3.87)	0.80 (3.60)	0.74 (3.75)	0.83 (3.49)	0.92 (4.12)	0.94 (4.10)	0.67 (3.10)	<0.001
Health score[a]	0.03 (3.64)	0.19 (4.07)	0.06 (3.79)	0.13 (4.24)	0.25 (4.25)	0.31 (4.29)	0.26 (3.91)	0.06 (3.58)	<0.001
Sleep duration[a]	7.18 (0.92)	7.19 (0.96)	7.15 (0.96)	7.16 (0.94)	7.12 (0.94)	7.16 (1.00)	7.13 (1.00)	7.20 (0.94)	<0.001
BMI[a]	26.38 (4.24)	26.57 (4.32)	26.81 (4.74)	25.63 (4.19)	26.59 (4.61)	27.61 (4.80)	27.78 (5.20)	24.70 (3.89)	<0.001
Ethnicity white, n (%)	32878 (97.1)	15010 (96.6)	14204 (97.0)	11926 (94.1)	3219 (96.3)	28571 (97.5)	6051 (95.9)	3632 (93.8)	<0.001
BMR[a]	1532.65 (256.79)	1571.14 (259.22)	1473.18 (240.50)	1491.13 (243.03)	1519.59 (248.15)	1575.71 (267.83)	1608.08 (276.47)	1444.43 (222.39)	<0.001
Overall health rating, n (%)									<0.001
Excellent	8115 (24.0)	3336 (21.5)	3440 (23.5)	2922 (23.0)	749 (22.4)	5178 (17.7)	1119 (17.7)	965 (24.9)	
Fair	4697 (13.9)	2541 (16.4)	2053 (14.0)	1848 (14.6)	504 (15.1)	5629 (19.2)	1238 (19.6)	518 (13.4)	
Good	20334 (60.1)	9205 (59.2)	8795 (60.1)	7594 (59.9)	1996 (59.7)	17410 (59.4)	3695 (58.6)	2296 (59.3)	
Poor	645 (1.9)	430 (2.8)	326 (2.2)	276 (2.2)	91 (2.7)	1041 (3.6)	243 (3.9)	78 (2.0)	
Smoking status, n (%)									<0.001
Previous	12302 (36.3)	5518 (35.5)	5444 (37.2)	4389 (34.6)	1279 (38.3)	10296 (35.1)	2216 (35.1)	1371 (35.4)	
Current	2043 (6.0)	1671 (10.8)	780 (5.3)	621 (4.9)	187 (5.6)	2215 (7.6)	632 (10.0)	199 (5.1)	
Never	19512 (57.6)	8348 (53.7)	8412 (57.5)	7667 (60.5)	1876 (56.1)	16798 (57.3)	3460 (54.9)	2302 (59.5)	
Alcohol drinker status, n (%)									<0.001
Previous	671 (2.0)	364 (2.3)	450 (3.1)	490 (3.9)	119 (3.6)	937 (3.2)	250 (4.0)	179 (4.6)	
Current	32494 (96.0)	14769 (95.1)	13811 (94.4)	11772 (92.9)	3110 (93.1)	27353 (93.3)	5832 (92.5)	3540 (91.4)	
Never	692 (2.0)	404 (2.6)	375 (2.6)	415 (3.3)	113 (3.4)	1019 (3.5)	226 (3.6)	153 (4.0)	
Physical activity (IPAQ), n (%)									<0.001
High	15101 (44.6)	6822 (43.9)	7061 (48.2)	6041 (47.7)	1638 (49.0)	12844 (43.8)	2874 (45.6)	1880 (48.6)	
Moderate	12658 (37.4)	5634 (36.3)	5163 (35.3)	4632 (36.5)	1178 (35.2)	10316 (35.2)	2186 (34.7)	1428 (36.9)	
Low	6098 (18.0)	3081 (19.8)	2412 (16.5)	2004 (15.8)	526 (15.7)	6149 (21.0)	1248 (19.8)	564 (14.6)	
Cardiovascular disease, n (%)	9625 (28.4)	4625 (29.8)	3988 (27.2)	3366 (26.6)	883 (26.4)	9322 (31.8)	1833 (29.1)	1021 (26.4)	<0.001
Hypertension, n (%)	8934 (26.4)	4167 (26.8)	3784 (25.9)	2806 (22.1)	817 (24.4)	9183 (31.3)	1659 (26.3)	729 (18.8)	<0.001
Diabetes, n (%)	1920 (5.7)	900 (5.8)	825 (5.6)	597 (4.7)	186 (5.6)	2583 (8.8)	549 (8.7)	136 (3.5)	<0.001
Nutrients intake									
Energy intake (MJ/day) [a]	7182.68 (1877.67)	7791.00 (2105.26)	7018.89 (1783.05)	7325.33 (1854.73)	7747.10 (1994.42)	7219.14 (1926.45)	7838.43 (2115.05)	7999.32 (2049.27)	<0.001
Energy density (kJ/g) [a]	6.37 (1.42)	7.09 (1.45)	5.65 (1.26)	6.18 (1.32)	6.06 (1.41)	6.69 (1.48)	7.14 (1.52)	6.15 (1.28)	<0.001
Englyst fiber (g/day) [a]	17.43 (5.41)	16.90 (5.43)	18.97 (6.23)	20.23 (6.16)	22.01 (7.01)	16.50 (5.37)	17.93 (5.85)	22.54 (6.98)	<0.001
Free sugar (g/day) [a]	54.42 (26.63)	71.11 (36.37)	49.87 (25.74)	58.36 (28.82)	57.12 (30.03)	65.60 (34.84)	68.82 (36.22)	57.28 (28.08)	<0.001
Protein (g/day) [a]	81.48 (20.50)	82.48 (21.82)	81.59 (21.10)	77.37 (20.38)	82.83 (22.02)	79.76 (20.54)	82.92 (22.87)	79.71 (22.18)	<0.001

Saturated fatty acids (g/day) [a]	26.75 (10.41)	31.79 (11.71)	24.35 (9.28)	25.21 (9.95)	25.10 (9.25)	27.13 (10.44)	30.57 (11.33)	28.77 (10.91)	<0.001
Sodium (g/day) [a]	1907.35 (663.61)	2126.02 (719.01)	1859.70 (638.26)	1853.51 (600.99)	2138.10 (692.22)	1955.99 (667.46)	2242.87 (726.65)	2014.57 (648.44)	<0.001
Main food groups (g/day)									
High fat cheese[a]	15.96 (17.15)	16.92 (17.93)	13.50 (15.42)	15.21 (16.92)	14.19 (15.15)	12.09 (14.50)	15.21 (16.57)	18.24 (17.87)	<0.001
Red meat[a]	42.04 (42.38)	43.90 (43.50)	36.87 (40.42)	29.61 (37.76)	21.05 (32.12)	45.23 (44.99)	35.79 (40.94)	18.56 (30.47)	<0.001
Vegetables[a]	182.39 (122.35)	169.02 (117.69)	250.20 (167.98)	205.31 (138.00)	243.15 (171.13)	161.69 (116.25)	157.66 (118.88)	250.52 (163.66)	<0.001
Fresh fruit[a]	197.14 (141.85)	158.84 (122.64)	245.28 (171.23)	211.20 (140.88)	240.68 (169.22)	176.95 (137.92)	167.27 (135.14)	235.03 (150.81)	<0.001
Whole grains[a]	4.56 (15.58)	0.00 (0.20)	19.29 (39.42)	25.91 (45.46)	19.63 (40.49)	0.12 (2.44)	14.83 (32.19)	23.16 (42.86)	<0.001
Coffee tea[a]	802.38 (321.50)	817.11 (308.55)	803.70 (330.30)	795.62 (336.20)	794.43 (332.70)	793.31 (326.49)	769.61 (332.19)	814.58 (343.80)	<0.001
Alcoholic drinks[a]	320.88 (435.29)	362.31 (493.33)	175.20 (225.38)	182.47 (256.61)	205.78 (280.79)	222.22 (318.40)	285.77 (428.64)	118.65 (152.36)	<0.001
SSBs and other sugary drinks[a]	30.45 (85.42)	23.52 (68.20)	100.33 (202.49)	29.74 (84.88)	166.50 (290.47)	118.67 (237.24)	162.02 (282.91)	0.42 (5.60)	<0.001
Water[a]	532.93 (377.96)	453.94 (356.11)	589.98 (386.45)	568.01 (392.55)	565.69 (387.12)	441.65 (344.49)	461.58 (356.55)	601.02 (399.93)	<0.001
High fat condiments[a]	13.22 (15.84)	14.92 (15.98)	14.58 (15.76)	12.23 (14.46)	12.35 (13.66)	13.48 (15.86)	12.59 (14.31)	12.77 (14.11)	<0.001
Milk based desserts[a]	21.65 (34.88)	25.60 (39.28)	20.59 (33.79)	23.07 (37.27)	21.67 (34.95)	30.38 (44.95)	24.68 (37.91)	23.09 (37.49)	<0.001
Chocolate confectionery[a]	8.29 (12.85)	14.34 (22.97)	8.64 (13.69)	10.68 (16.89)	13.01 (22.84)	15.87 (24.40)	16.46 (24.65)	9.59 (14.79)	<0.001
Butter and other animal-fat spreads[a]	5.94 (8.96)	7.44 (9.72)	3.37 (5.74)	4.21 (7.18)	0.75 (2.15)	4.51 (7.69)	6.41 (9.70)	5.33 (8.23)	<0.001

Supplementary Table S27 | Baseline characteristics of participants by latent profile (N = 119,709)

TDI Townsend deprivation index, IPAQ International Physical Activity Questionnaire, SSBs Sugar-sweetened beverages, BMR Basal metabolic rate, G Groups, DP Dietary Pattern, 1-4 Quintile 1-4

*ANOVA or χ2 test where appropriate

[a]Mean (SD)

Supplementary Table S28

Metabolic syndrome component	Harmonized criteria (2009)<br>*Three or more of the following:*	Field code description
**Elevated waist circumference**	Elevated waist circumference: ≥ 102 cm in males and ≥ 88 cm in females	Waist circumference
**Elevated triglycerides**	≥ 150 mg/dL (1.7 mmol/L)	Triglycerides Blood Biochemistry;
**Elevated blood pressure**	Systolic: ≥130 mmHg and/or Diastolic ≥85 mmHg*, or antihypertensive medication use<br><br>*In UKB, BP was measured twice by trained nurses after participants had been at rest for at least 5 minutes. Individual systolic and diastolic BP measurements were averaged within a visit. Automated BP readings were the preferred source of data. However, if this was unavailable, manual readings were used	Systolic BP - automated reading, Diastolic BP - automated reading;<br><br>Systolic BP - manual reading, Diastolic BP - manual reading<br><br>ATC Codes starting with: C02, C03, C07, C08, C09
**Elevated blood glucose**	≥100 mg/dL (≥5.6 mmol/L) †, or drug treatment for elevated glucose level<br><br>†HbA1c used as a proxy indicator, with cut-offs based on the recommendations of the American Diabetes Association: HbA1c ≥5.7%	Glycated haemoglobin (hbA1C)<br><br>ATC Codes starting with: A10
**Reduced HDL cholesterol**	Males: <40 mg/dL (1.0 mmol/L); Females: <50 mg/dL (1.3 mmol/L), or lipid-modifying medications	HDL cholesterol<br><br>ATC Codes starting with: C10

Supplementary Table S28 | Variables and medication codes used to define MetS in the UKB cohort

UKB = UK Biobank, ATC = Anatomical Therapeutic Chemical, HDL = High-density lipoprotein, BP = Blood pressure, HbA1c = Glycated haemoglobin

Supplementary Table S29

UKB Drug Category	UKB Drug Name	UKB Code	ATC Code
	MEDICATIONS FOR REDUCED HDL CHOLESTEROL		
atorvastatin	Atorvastatin	1141146234	C10AA05
lipitor 10mg tablet	Atorvastatin	1141146138	C10AA05
rosuvastatin	Rosuvastatin	1141192410	C10AA07
crestor 10mg tablet	Rosuvastatin	1141192414	C10AA07
simvastatin	Simvastatin	1140861958	C10AA01
simvador 10mg tablet	Simvastatin	1141188146	C10AA01
zocor 10mg tablet	Simvastatin	1140881748	C10AA01
pravastatin	Pravastatin	1140888648	C10AA03
lipostat 10mg tablet	Pravastatin	1140861970	C10AA03
ezetimibe	Ezetimibe	1141192736	C10AX09
ezetrol 10mg tablet	Ezetimibe	1141192740	C10AX09
fenofibrate	Fenofibrate	1140861954	C10AB05
lipantil micro 67mg capsule	Fenofibrate	1141162544	C10AB05
supralip 160mg m/r tablet	Fenofibrate	1141172214	C10AB05
fluvastatin	Fluvastatin	1140888594	C10AA04
lescol 20mg capsule	Fluvastatin	1140864592	C10AA04
gemfibrozil	Gemfibrozil	1140861856	C10AB04
omacor 1g capsule	Omega-3-Acid Ethyl Esters	1141181868	C10AX06
bezafibrate	Bezafibrate	1140861924	C10AB02
questran 4g/sachet powder	Cholestyramine	1140861936	C10AC01
colestyramine	Cholestyramine	1140909780	C10AC01
ciprofibrate	Ciprofibrate	1140862026	C10AB08
bezalip-mono 400mg m/r tablet	Bezafibrate	1140861928	C10AB02
bezalip 200mg tablet	Bezafibrate	1140861926	C10AB02
niacin	Niacin	1140910670	C04AC01 |C10AD02
niaspan 500mg m/r tablet	Niacin	1141188546	C10AD02
nicotinic acid product	Niacin	1140861868	C04AC01 |C10AD02
cholestyramine	Cholestyramine	1140865576	C10AC01
fibrazate xl 400mg m/r tablet	Bezafibrate	1141201306	C10AB02
colestyramine product	Cholestyramine	1141180734	C10AC01
bezafibrate product	Bezafibrate	1141157260	C10AB02
modalim 100mg tablet	Ciprofibrate	1140862028	C10AB08
zimbacol xl 400mg m/r tablet	Bezafibrate	1141171548	C10AB02
colestipol	Colestipol	1140888590	C10AC02
colestid 5g/sachet granules	Colestipol	1140861848	C10AC02
acipimox	Acipimox	1140861892	C10AD06

colestyramine+aspartame 4g/sachet powder	Cholestyramine	1141180722	C10AC01
MEDICATIONS FOR ELEVATED BLOOD PRESSURE			
hydralazine	Hydralazine	1140888686	C02DB02
moxonidine	Moxonidine	1140928284	C02AC05
physiotens 200micrograms tablet	Moxonidine	1140928290	C02AC05
minoxidil	Minoxidil	1140860532	C02DC01 |D11AX01
doxazosin	Doxazosin	1140879778	C02CA04
methyldopa	Methyldopa	1140860470	C02AB
cardura 1mg tablet	Doxazosin	1140860690	C02CA04
doxadura 1mg tablet	Doxazosin	1141194372	C02CA04
clonidine	Clonidine	1140883468	C02AC01 |N02CX02 |S01EA04
indoramin	Indoramin	1140879782	C02CA02
prazosin	Prazosin	1140879794	C02CA01
hypovase 500mcg tablet	Prazosin	1140860580	C02CA01
indapamide	Indapamide	1140866078	C03BA11
natrilix sr 1.5mg m/r tablet	Indapamide	1141146378	C03BA11
hydrochlorothiazide	Hydrochlorothiazide	1140866162	C03AA03
furosemide	Furosemide	1140909708	C03CA01
frusemide	Furosemide	1140866116	C03CA01
spironolactone	Spironolactone	1140866236	C03DA01
spirolone 25mg tablet	Spironolactone	1140866318	C03DA01
chlortalidone	Chlorthalidone	1140909706	C03BA04
eplerenone	Eplerenone	1141201244	C03DA04
hygroton 50mg tablet	Chlorthalidone	1140866146	C03BA04
metolazone	Metolazone	1140866092	C03BA08
bendroflumethiazide	Bendroflumethiazide	1141194794	C03AA01
bendrofluazide	Bendroflumethiazide	1140866122	C03AA01
co-amilofruse	Amiloride |Furosemide	1140923402	C03EB01
bumetanide	Bumetanide	1140866280	C03CA02
co-amilozide	Amiloride |Hydrochlorothiazide	1140923276	C03EA01
amiloride	Amiloride	1140888512	C03DB01
bendroflumethiazide+potassium 2.5mg/7.7mmol m/r tablet	Bendroflumethiazide |Potassium	1141194800	C03AB01
frumil tablet	Amiloride |Furosemide	1140866406	C03CA01
bendrofluazide+potassium 2.5mg/7.7mmol m/r tablet	Bendroflumethiazide |Potassium	1140866450	C03AB01

bzt - bendrofluazide	Bendroflumethiazide	1140910442	C03AA01
dyazide tablet	Triamterene |Hydrochlorothiazide	1140866402	C03EA01
moduretic tablet	Amiloride |Hydrochlorothiazide	1140866420	C03EA01
navispare tablet	Amiloride |Cyclopenthiazide	1140866352	C03EA07
co-triamterzide	Triamterene |Hydrochlorothiazide	1140923272	C03EA01
torasemide	Torasemide	1140888496	C03CA04
moduret 25 tablet	Amiloride |Hydrochlorothiazide	1140866416	C03EA01
burinex a tablet	Bumetanide	1140866356	C03CA02
xipamide	Xipamide	1140866108	C03BA10
cyclopenthiazide	Cyclopenthiazide	1140866156	C03AA07
bisoprolol	Bisoprolol	1140879760	C07AB07
cardicor 1.25mg tablet	Bisoprolol	1141171152	C07AB07
atenolol	Atenolol	1140866738	C07AB03
tenormin 25 tablet	Atenolol	1140866756	C07AB03
metoprolol	Metoprolol	1140879818	C07AB02
carvedilol	Carvedilol	1140909368	C07AG02
propranolol	Propranolol	1140879842	C07AA05
timolol	Timolol	1140879866	C07AA06 |S01ED01
half-inderal la 80mg m/r capsule	Propranolol	1140866800	C07AA05
inderal 10mg tablet	Propranolol	1140866804	C07AA05
bedranol 10mg tablet	Propranolol	1140851556	C07AA05
half beta-prograne 80mg m/r capsule	Propranolol	1140866802	C07AA05
labetalol	Labetalol	1140879824	C07AG01
sotalol	Sotalol	1140879854	C07AA07
beta-cardone 40mg tablet	Sotalol	1140860304	C07AA07
co-tenidone	Chlorthalidone |Atenolol	1140923336	C07CB03
nebivolol	Nebivolol	1141164276	C07AB12
bisoprolol fumarate+hydrochlorothiazide 10mg/6.25mg tablet	Bisoprolol |Hydrochlorothiazide	1140864950	C07BB07
celiprolol	Celiprolol	1140879762	C07AB08
atenolol+bendroflumethiazide	Atenolol |Bendroflumethiazide	1141194810	C07BB03
nebilet 5mg tablet	Nebivolol	1141164280	C07AB12
propranolol hydrochloride+bendrofluazide 80mg/2.5mg capsule	Bendroflumethiazide |Propranolol	1140860418	C07BA05

sotalol hydrochloride+hydrochlorothiazide 80mg/12.5mg tablet	Sotalol |Hydrochlorothiazide	1140860332	C07BA07
tenoret 50 tablet	Chlorthalidone |Atenolol	1140860324	C07CB03
carteolol	Carteolol	1140879822	C07AA15 |S01ED05
betaxolol	Betaxolol	1140879758	C07AB05 |S01ED02
atenolol+bendrofluazide	Atenolol |Bendroflumethiazide	1141146126	C07BB03
tenif capsule	Atenolol |Nifedipine	1140860358	C07FB03
metoprolol tartrate+chlorthalidone 100mg/12.5mg tablet	Metoprolol |Chlorthalidone	1140860308	C07CB02
tenoretic tablet	Chlorthalidone |Atenolol	1140860328	C07CB03
beta-adalat capsule	Atenolol |Nifedipine	1140860356	C07FB03
celectol 200mg tablet	Celiprolol	1140860498	C07AB08
acebutolol	Acebutolol	1140866724	C07AB04
oxprenolol	Oxprenolol	1140879830	C07AA02
prindolol	Pindolol	1140910614	C07AA17
kalten capsule	Atenolol |Amiloride |Hydrochlorothiazide	1140860398	C07DB01
nadolol	Nadolol	1140860192	C07AA12
pindolol	Pindolol	1140860292	C07AA03
atenolol+chlortalidone	Chlorthalidone |Atenolol	1141180778	C07BB03
amlodipine	Amlodipine	1140879802	C08CA01
istin 5mg tablet	Amlodipine	1140861202	C08CA01
amlostin 5mg tablet	Amlodipine	1141200400	C08CA01
nifedipine	Nifedipine	1140861088	C08CA05
adalat 5mg capsule	Nifedipine	1140861090	C08CA05
coracten sr 10mg m/r capsule	Nifedipine	1140861120	C08CA05
adalate 10mg capsule	Nifedipine	1140881702	C08CA05
adipine mr 10 m/r tablet	Nifedipine	1140923572	C08CA05
fortipine la40 m/r tablet	Nifedipine	1141145870	C08CA05
nifedipress mr 10 m/r tablet	Nifedipine	1141157140	C08CA05
tensipine mr 10 m/r tablet	Nifedipine	1140927940	C08CA05
verapamil	Verapamil	1140888510	C08DA01
securon 40mg tablet	Verapamil	1140866466	C08DA01
half securon sr 120mg m/r tablet	Verapamil	1140866460	C08DA01
univer 120mg m/r capsule	Verapamil	1140881692	C08DA01
vertab sr 240 m/r tablet	Verapamil	1141169710	C08DA01
diltiazem	Diltiazem	1140879806	C05AE03 |C08DB01
tildiem 60mg m/r tablet	Diltiazem	1140861128	C08DB01

adizem-60 m/r tablet	Diltiazem	1140861138	C08DB01
adizem-xl plus m/r capsule	Diltiazem	1140926780	C08DB01
dilzem sr 60mg long acting m/r capsule	Diltiazem	1140861166	C08DB01
slozem 120mg m/r capsule	Diltiazem	1140911698	C08DB01
angitil sr 90 m/r capsule	Diltiazem	1140917428	C08DB01
viazem xl 120mg m/r capsule	Diltiazem	1141151474	C08DB01
zemtard 120 xl m/r capsule	Diltiazem	1141167832	C08DB01
calcicard 60mg tablet	Diltiazem	1140851730	C08DB01
felodipine	Felodipine	1140888646	C08CA02
cardioplen xl 5mg m/r tablet	Felodipine	1141199858	C08CA02
vascalpha 5mg m/r tablet	Felodipine	1141190160	C08CA02
felendil xl 5mg m/r tablet	Felodipine	1141188836	C08CA02
plendil 2.5mg m/r tablet	Felodipine	1140928212	C08CA02
felotens xl 5mg m/r tablet	Felodipine	1141188152	C08CA02
neofel xl 5mg m/r tablet	Felodipine	1141200782	C08CA02
felogen xl 5mg m/r tablet	Felodipine	1141188576	C08CA02
cabren 2.5mg m/r tablet	Felodipine	1141187094	C08CA02
lercanidipine	Lercanidipine	1141153026	C08CA13
zanidip 10mg tablet	Lercanidipine	1141153032	C08CA13
lacidipine	Lacidipine	1140861276	C08CA09
diltiazem hcl+hydrochlorothiazide 150mg/12.5mg m/r capsule	Diltiazem |Hydrochlorothiazide	1140926778	C08GA
nicardipine	Nicardipine	1140879810	C08CA04
motens 2mg tablet	Lacidipine	1140861282	C08CA09
cardene 20mg capsule	Nicardipine	1140861176	C08CA04
valsartan	Valsartan	1141145660	C09CA03
diovan 40mg capsule	Valsartan	1141145668	C09CA03
losartan	Losartan	1140916356	C09CA01
cozaar 25mg tablet	Losartan	1141179974	C09CA01
perindopril	Perindopril	1140888560	C09AA04
coversyl 2mg tablet	Perindopril	1140860802	C09AA04
lisinopril	Lisinopril	1140860696	C09AA03
zestril 2.5mg tablet	Lisinopril	1140860714	C09AA03
irbesartan	Irbesartan	1141152998	C09CA04
aprovel 75mg tablet	Irbesartan	1141153006	C09CA04
enalapril	Enalapril	1140888552	C09AA02
innovace 2.5mg tablet	Enalapril	1140860776	C09AA02
fosinopril	Fosinopril	1140888556	C09AA09
ramipril	Ramipril	1140860806	C09AA05
telmisartan	Telmisartan	1141166006	C09CA07
micardis 20mg tablet	Telmisartan	1141172492	C09CA07

tritace 1.25mg tablet	Ramipril	1141188408	C09AA05
lopace 2.5mg capsule	Ramipril	1141199940	C09AA05
candesartan cilexetil	Candesartan	1141156836	C09CA06
amias 2mg tablet	Candesartan	1141156846	C09CA06
cilazapril	Cilazapril	1140860882	C09AA08
olmesartan	Olmesartan	1141193282	C09CA08
losartan potassium+hydrochlorothiazide 50mg/12.5mg tablet	Losartan |Hydrochlorothiazide	1141151016	C09DA01
olmetec 10mg tablet	Olmesartan	1141193346	C09CA08
trandolapril	Trandolapril	1140860904	C09AA10
eprosartan	Eprosartan	1141171336	C09CA02
captopril	Captopril	1140860750	C09AA01
quinapril	Quinapril	1140860728	C09AA06
lisinopril+hydrochlorothiazide 10mg/12.5mg tablet	Lisinopril |Hydrochlorothiazide	1140864952	C09BA03
coaprovel 150mg/12.5mg tablet	Hydrochlorothiazide |Irbesartan	1141172686	C09DA04
enalapril maleate+hydrochlorothiazide 20mg/12.5mg tablet	Enalapril |Hydrochlorothiazide	1140860790	C09BA02
irbesartan+hydrochlorothiazide 150mg/12.5mg tablet	Hydrochlorothiazide |Irbesartan	1141172682	C09DA04
cozaar-comp 50mg/12.5mg tablet	Losartan |Hydrochlorothiazide	1141151018	C09DA01
perindopril+indapamide	Perindopril |Indapamide	1141180592	C09BA04
zestoretic 10 tablet	Lisinopril |Hydrochlorothiazide	1140864618	C09BA03
coversyl plus 4mg/1.25mg tablet	Perindopril |Indapamide	1141180598	C09BA04
co-diovan 80mg/12.5mg tablet	Valsartan |Hydrochlorothiazide	1141201040	C09DA03
valsartan+hydrochlorothiazide 80mg/12.5mg tablet	Valsartan |Hydrochlorothiazide	1141201038	C09DA03
teveten 300mg tablet	Eprosartan	1141171344	C09CA02
micardisplus 40mg/12.5mg tablet	Telmisartan |Hydrochlorothiazide	1141187790	C09DA07
telmisartan+hydrochlorothiazide 40mg/12.5mg tablet	Telmisartan |Hydrochlorothiazide	1141187788	C09DA07
felodipine+ramipril	Ramipril |Felodipine	1141165470	C09BB05
imidapril hydrochloride	Imidapril	1141164148	C09AA16
innozide tablet	Enalapril |Hydrochlorothiazide	1140860784	C09BA02
gopten 500micrograms capsule	Trandolapril	1140860912	C09AA10
capozide tablet	Hydrochlorothiazide |Captopril	1140881714	C09BA01
capoten 12.5mg tablet	Captopril	1140860758	C09AA01

carace 10 plus tablet	Lisinopril |Hydrochlorothiazide	1140864910	C09BA03
triapin mite 2.5mg/2.5mg tablet	Ramipril |Felodipine	1141165476	C09BB05
accupro 5mg tablet	Quinapril	1140881706	C09AA06
MEDICATIONS FOR ELEVATED BLOOD GLUCOSE			
metformin	Metformin	1140884600	A10BA02
glucophage 500mg tablet	Metformin	1140874686	A10BA02
insulin product	Insulin	1140883066	A10A
gliclazide	Gliclazide	1140874744	A10BB09
glyclizide	Gliclazide	1140910566	A10BB09
diamicron 80mg tablet	Gliclazide	1140874746	A10BB09
glimepiride	Glimepiride	1141152590	A10BB12
amaryl 1mg tablet	Glimepiride	1141156984	A10BB12
pioglitazone	Pioglitazone	1141171646	A10BG03
actos 15mg tablet	Pioglitazone	1141171652	A10BG03
glibenclamide	Glyburide	1140874718	A10BB01
repaglinide	Repaglinide	1141168660	A10BX02
rosiglitazone	Rosiglitazone	1141177600	A10BG02
rosiglitazone 1mg / metformin 500mg tablet	Metformin |Rosiglitazone	1141189090	A10BD03
avandamet 1mg / 500mg tablet	Metformin |Rosiglitazone	1141189094	A10BD03
glipizide	Glipizide	1140874646	A10BB07
avandia 4mg tablet	Rosiglitazone	1141177606	A10BG02
tolbutamide	Tolbutamide	1140874674	A10BB03 |V04CA01
acarbose	Acarbose	1140868902	A10BF01
nateglinide	Nateglinide	1141173882	A10BX03

Supplementary Table S29 | Detailed description of UKB medication codes (mapped to ATC codes) used to define MetS components

Supplementary Table S7

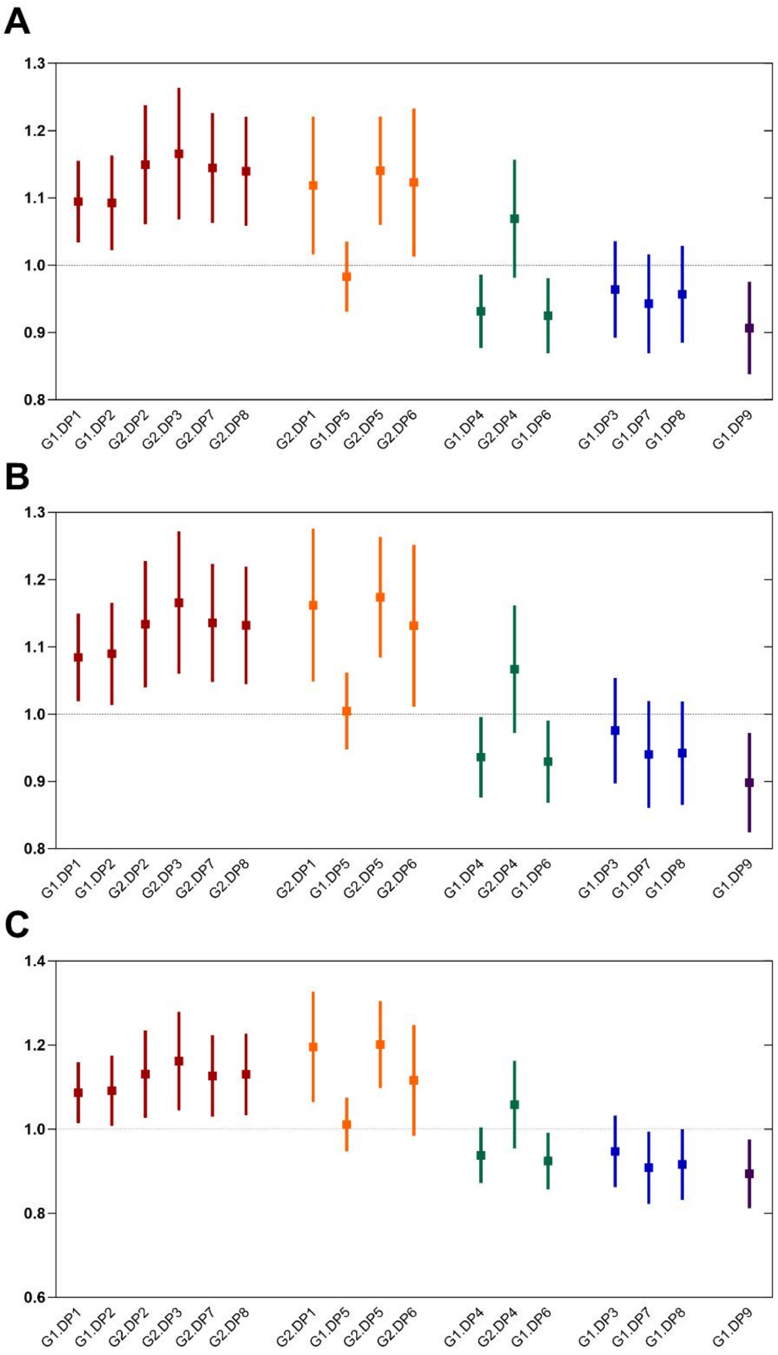

Supplementary Figure S7 | Sensitivity analysis.

Figure A presents results after excluding participants who developed renal cysts within 2 years post-baseline. Figure B shows results adjusted for metabolic syndrome covariates as per Supplementary 28 & 29. Figure C depicts results after excluding participants with baseline eGFR < 60 mL/min/1.73 m^2. The dietary patterns are color-coded as follows: red for Lipid-Rich, Calorically Dense Diet; orange for Hyper Glycemic, Fiber-Deficient Diet; green for Micronutrient-Abundant, Low-Lipid Diet; blue for Mineral-Rich, Moderate-Fat Diet; and purple for Fiber-Enriched, Lipid-Conservative Diet.
